# Cell therapies in the clinic

**DOI:** 10.1002/btm2.10214

**Published:** 2021-02-26

**Authors:** Lily Li‐Wen Wang, Morgan E. Janes, Ninad Kumbhojkar, Neha Kapate, John R. Clegg, Supriya Prakash, Mairead K. Heavey, Zongmin Zhao, Aaron C. Anselmo, Samir Mitragotri

**Affiliations:** ^1^ John A. Paulson School of Engineering & Applied Sciences Harvard University Cambridge Massachusetts USA; ^2^ Wyss Institute for Biologically Inspired Engineering Boston Massachusetts USA; ^3^ Harvard‐MIT Division of Health Sciences and Technology, Massachusetts Institute of Technology Cambridge Massachusetts USA; ^4^ Division of Pharmacoengineering and Molecular Pharmaceutics, Eshelman School of Pharmacy University of North Carolina at Chapel Hill Chapel Hill North Carolina USA

**Keywords:** blood cell, cell, cell therapy, clinical translation, clinical trials, microbes, stem cell, T cell

## Abstract

Cell therapies have emerged as a promising therapeutic modality with the potential to treat and even cure a diverse array of diseases. Cell therapies offer unique clinical and therapeutic advantages over conventional small molecules and the growing number of biologics. Particularly, living cells can simultaneously and dynamically perform complex biological functions in ways that conventional drugs cannot; cell therapies have expanded the spectrum of available therapeutic options to include key cellular functions and processes. As such, cell therapies are currently one of the most investigated therapeutic modalities in both preclinical and clinical settings, with many products having been approved and many more under active clinical investigation. Here, we highlight the diversity and key advantages of cell therapies and discuss their current clinical advances. In particular, we review 28 globally approved cell therapy products and their clinical use. We also analyze >1700 current active clinical trials of cell therapies, with an emphasis on discussing their therapeutic applications. Finally, we critically discuss the major biological, manufacturing, and regulatory challenges associated with the clinical translation of cell therapies.

## 1 INTRODUCTION

Cell therapies represent a major frontier and paradigm shift in biotechnology. In contrast to conventional therapeutic modalities, cell therapies are living and can dynamically respond to biological cues to attack malignancies, regenerate tissues, restore impaired or lost biological functions, or otherwise augment the body's own capability to fight disease (e.g., vaccination, immunomodulation).^1^ Cell therapies hold exceptional promise particularly because cells can function in ways that conventional small molecules and biologics cannot. Uniquely, living cells can simultaneously respond to both systemic and local chemical, physical, and biological cues, readily breach biological barriers,^1^ molecularly target and interact with specific cell types and tissues,^2^ and serve as a platform for additional therapeutic functions (e.g., cellular hitchhiking, genetic engineering).^3^, ^4^, ^5^ In this review, we have identified 28 cell therapy products approved for clinical use and 1705 active clinical trials employing cells for therapeutic purposes. We provide a snapshot of the clinical landscape of cell therapies by: (i) highlighting these approved products; (ii) summarizing and reviewing these current clinical trials based on their cell type, indication, source, and phase; and (iii) discussing the challenges associated with clinical translation. For mammalian cell‐based therapies, we restricted our analysis to applications where cells are administered as a single cell‐suspension (i.e., exclusion of tissue scaffolds and whole blood transplants). Our discussion is mainly focused on blood cells (T cells, natural killer [NK] cells, red blood cells [RBCs], dendritic cells [DCs], mononuclear cells, and platelets) and stem cells. We also provide an update on the clinical status of microbe therapeutics, which have recently emerged as a promising class of cell therapies for the treatment of infections and cancer.

## 2 CLINICAL LANDSCAPE

The global market for cell therapy is predominantly shared by stem cells and tissue‐specific cells (e.g., skin cells, chondrocytes), followed by blood cells.^6^, ^7^, ^8^ Current approved stem‐cell therapies include hematopoietic stem cells (HSCs), mesenchymal stem cells (MSCs), and to a lesser extent limbal stem cells (LSCs). HSC products are predominantly approved for the treatment of blood disorders. MSC therapies are indicated for a broad variety of diseases, including cardiovascular diseases, graft versus host diseases (GvHD), degenerative disorders, and inflammatory bowel diseases. The single LSC product is approved for LSC deficiency. Distinct from stem cell products, terminally differentiated tissue‐specific cells are mainly used for regenerative medicine and tissue engineering applications, such as autologous skin cells (i.e., keratinocytes, fibroblasts, and melanocytes) for the treatment of thermal burns,^9^ bi‐layers of living cellular skin substitute for venous leg ulcers and diabetic foot ulcers,^10^ and autologous chondrocyte scaffolds for repair of cartilage defects.^11^ These tissue‐specific cell therapies are beyond the scope of this review because they are mostly applied as tissue scaffolds instead of as single‐cell suspension and have been extensively reviewed elsewhere.^12^, ^13^, ^14^ The third group of cell therapies consist of blood cells, including leukocytes, RBCs, and platelets; however, only T cells and DCs have been approved as therapeutic products in the market to date. Most approved T cell products are chimeric antigen receptor (CAR)‐T therapies for hematologic malignancies, whereas DC products are used as vaccines for solid cancers. We should also note that RBCs and platelets, while not associated with a specific product, are widely used in clinical settings for blood transfusions.^15^ In addition, the cell source of these approved products can be originated either from the patients themselves (autologous) or from the other donors (allogeneic).

**FIGURE 1:**
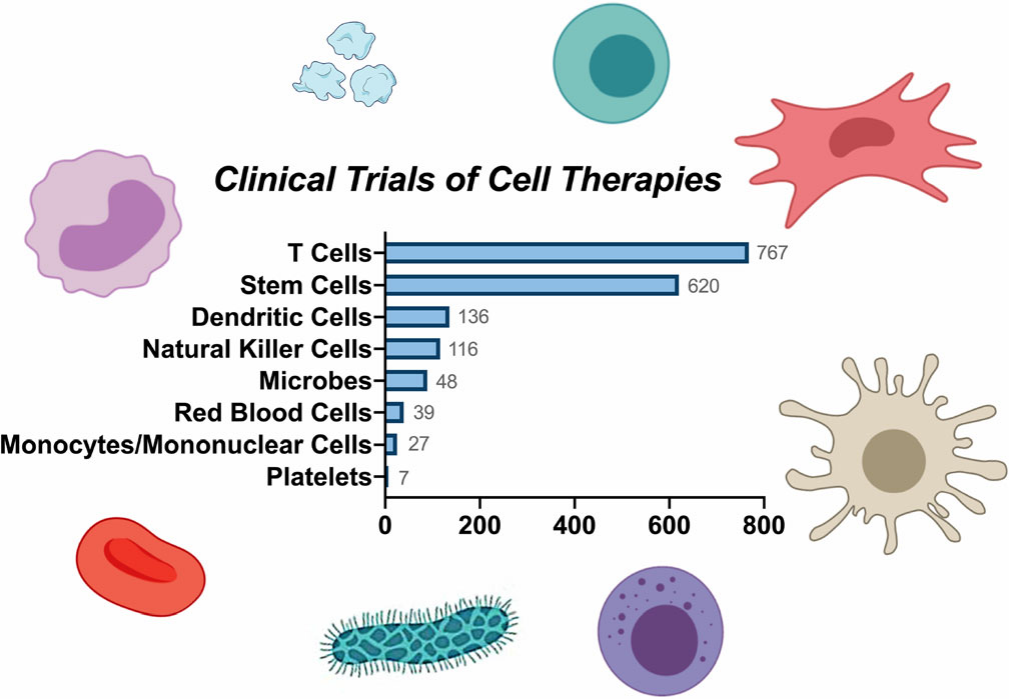
Various types of cell therapies in clinical trials. T cells dominate the current clinical studies of cell therapies, followed by stem cells, dendritic cells, natural killer cells, microbes, red blood cells, mononuclear cells, and platelets

Although stem cells and tissue‐specific cells account for the vast majority of approved cell therapies in the current market, blood cells have emerged as the dominant cell type that is being developed and evaluated in clinical trials. Just 5 years ago, the number of trials for MSCs alone was greater than the number of trials for all lymphocytes and DCs combined.^14^ Currently, T‐cell trials individually outnumber *all* stem cell trials, and far exceed those for tissue‐specific cells. This ongoing shift is driven primarily by the recent clinical success of CAR‐T therapy, which is in turn a product of major breakthroughs in our understanding of how immune modulatory approaches can be used to treat disease.^16^, ^17^, ^18^, ^19^ In light of this trend, we collected and analyzed clinical trials that use blood cells, with additional focus on stem cells delivered as single‐cell suspensions, and microbes (including non‐single‐cell suspension dosage forms), which have recently emerged as promising agents for the treatment of infections and cancer. Specifically, we identified the trials on clinicaltrials.gov by searching for each cell type (Figure 1) with the following key words (listed in parentheses) in the “Intervention/treatment” category: T cells (“T cell”; system also automatically searched for “T lymphocyte”), stem cells (“stem cell”; system also automatically searched for “progenitor cell”), natural killer cells (“natural killer,” “NK”), dendritic cells (“dendritic cell,” “DC”; system also automatically searched for “antigen presenting cell” and “cellular”), monocytes (“monocyte”; system also automatically searched for “monocytic”), macrophages (“macrophage”), bone marrow‐derived mononuclear cells (“bone marrow‐derived mononuclear cell”), peripheral blood mononuclear cells (“peripheral blood mononuclear cell”; system also automatically searched for “peripheral blood,” “blood,” “whole blood”), red blood cells (“red blood cell”; system also automatically searched for “erythrocytes,” “red cells,” “whole blood,” “RBC count,” and “blood corpuscles”), platelets (“platelet”; system also automatically searched for “thrombocyte”), and microbes (“live biotherapeutic,” “bacteria,” “consortia”). In the “Status” category under “Recruitment,” we selected trials with statuses of not yet recruiting, recruiting, enrolling by invitation, and active/not recruiting. The collected data capture the clinical landscape as of August 2020. We then manually filtered the trials to exclude entries that mentioned the cell types of interest but did not use them as therapeutic interventions. Finally, we excluded long‐term follow‐up studies that did not involve re‐administration of the therapy. A list of abbreviations used through this manuscript is shown in Table S1.

**FIGURE 2:**
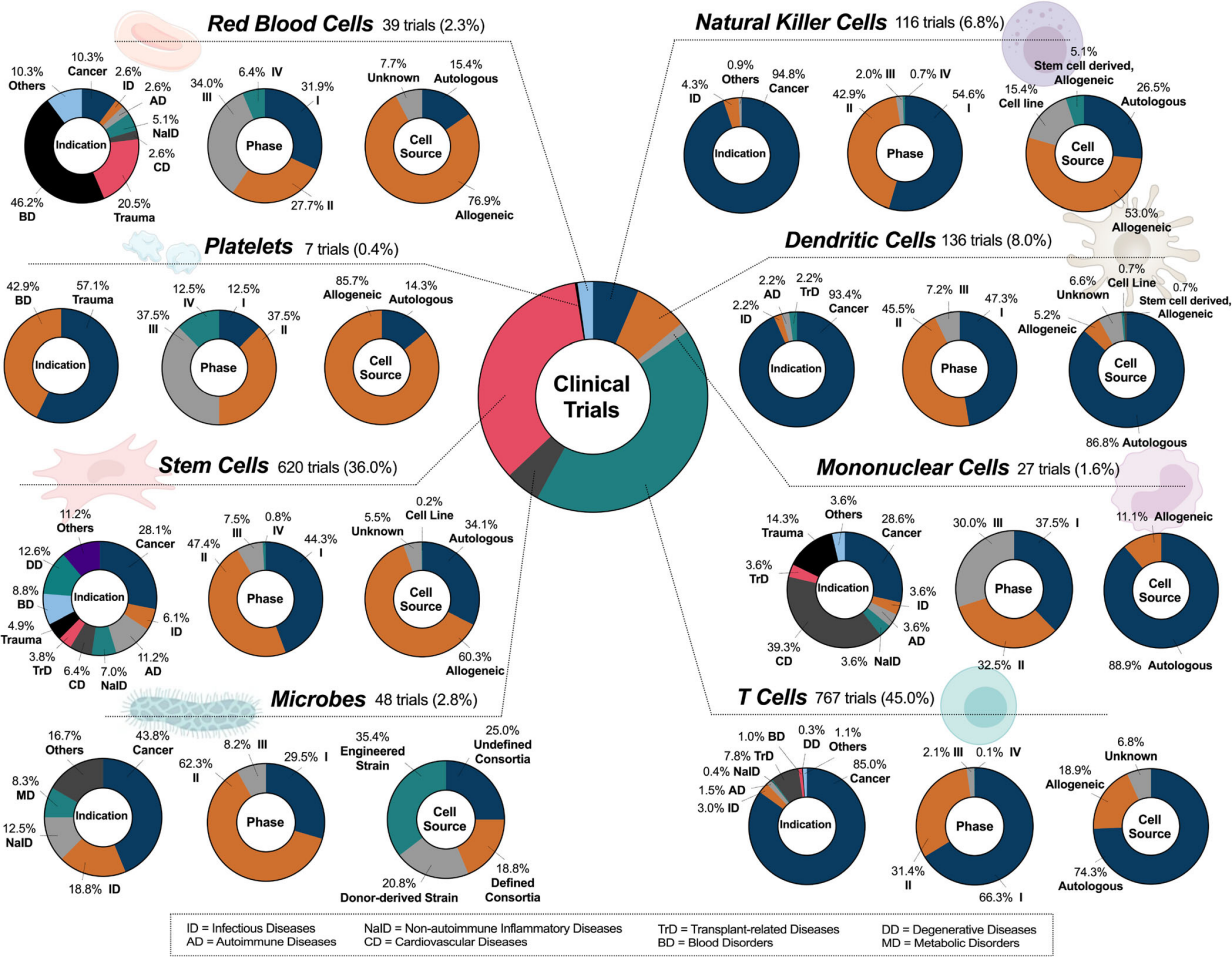
Current landscape of cell therapies in clinical trials. In this review, all clinical trials that include blood cells and stem cells delivered as a suspension were cataloged. Trials using microbes (delivered via various routes and dosage forms) were also included, as they represent an emerging class of therapies for similar applications. The relevant cell types include T cells, NK cells, mononuclear cells, DCs, RBCs, platelets, stem cells, and microbes. Tissue‐specific cells were excluded from the analysis. The total number of trials identified for each cell type is displayed in the figure, however the sum of these trials for all cell types (1760) exceeds the total number of analyzed trials (1705) because some trials use two or more cell therapies in combination. For phase classification, dual‐phase trials (e.g., Phase 1/2) were counted as both Phase 1 and 2. Eleven broad indications were identified for the purpose of trial classification (i.e., cancer, infectious diseases, autoimmune diseases, nonautoimmune inflammatory diseases, cardiovascular diseases, transplant‐related diseases, trauma, blood disorders, degenerative diseases, metabolic disorders, etc.), with relevant abbreviations listed in the box at the bottom of the figure. Because some trials are used to treat more than one of these conditions, the total number of indications used to generate each pie chart exceeds the total number of trials for each cell type

A total of 1705 unique, active cell therapy clinical trials have been identified and categorized according to cell type, general indication, trial phase, and cell source (Figure 2). Among only leukocytes, T cells account for the largest portion of all current trials (767/1705, 45%), followed by DCs (136/1705, 8%), NK cells (116/1705, 7%), and the remaining mononuclear cells (27/1705, 2%). It is unsurprising that the main indication of T cells, DCs, and NK cells is cancer (85% in T cells; 93% in DCs; 95% in NK cells), as they play major roles in anti‐cancer immunity. T cells are adaptive immune cells capable of directly eliminating mutated or infected host cells, activating other immune cells, and producing cytokines to regulate immune responses.^20^ NK cells are innate immune cells that destroy tumor cells and virally infected cells via release of lytic molecules from granules and rapid production of pro‐inflammatory cytokines.^21^ DCs are professional antigen‐presenting cells (APCs) that regulate adaptive immune cells by delivering antigens to draining lymph nodes and presenting them to cytotoxic and helper T cells.^22^ In the case of cancer treatment, T and NK cells are employed as cytotoxic agents, while DCs primarily serve as cancer vaccines. From the perspective of cell source, autologous cells are mainly used in T cell (74%) and DC (87%) therapy, as allogeneic cells increase the risk of allograft rejection (recipient cells against donor cells) or, more considerably, GvHD (donor cells against recipient cells).^23^

While the aforementioned leukocytes are mainly indicated for the treatment of cancer, the remaining mononuclear cells are used mostly for cardiovascular diseases (39%) and cancer (29%). For the purpose of this review, we refer to mononuclear cells as belonging to one of the following cell populations: monocytes, macrophages, bone marrow‐derived mononuclear cells (BMMCs), or peripheral blood mononuclear cells (PBMCs). Monocytes are circulatory cells of the innate immune system that extravasate into tissue in response to inflammation, infection, or injury.^24^ Once in the tissue they terminally differentiate into macrophages, which are tissue‐resident innate immune cells that (i) phagocytose dead cells, debris, and foreign materials/pathogens, (ii) modulate innate immune responses, and (iii) maintain homeostatic growth, repair, and metabolism.^25^ BMMCs are a heterogeneous group of cells composed of lymphoid cells, myeloid cells, HSCs, and MSCs. They have major clinical applications in cardiovascular tissue regeneration due to their ability to differentiate into various lineages.^26^ Similar to BMMCs, PBMCs also contain a variety of cells including lymphocytes, monocytes, and a small percentage of DCs and are mainly indicated for cancers. Currently, there are no Phase 4 trials employing mononuclear cells, with similar representations across Phases 1–3. In addition, autologous mononuclear cells are employed almost exclusively (89%), likely to reduce the risk of graft rejection and GvHD.

The remaining blood cells, RBCs and platelets, are used as cell therapies for treatment of blood disorders and in trauma care via blood transfusions, and account for 2% (39/1705) and <0.4% (7/1705) of all current cell therapy trials, respectively (Figure 2). Typically, they are used to replenish lost or dysfunctional cells to maintain homeostasis in the body. The RBC is a critical transporter of oxygen and nutrients to tissues as well as an inter‐organ communicator, with additional roles in the regulation of pH, redox homeostasis, and molecular metabolism.^27^ Hence, loss of RBC integrity and/or number can lead to severe pathologies and heighten the incidence of vascular disease. Similarly, the platelet serves as a key element in blood vessels by regulating hemostasis under normal conditions and thrombosis upon vascular damage.^28^ Thrombocytopenia (i.e., platelet deficiency) that results from either trauma or blood disorders can lead to hemorrhage in tissues or uncontrolled bleeding of wounds. Both RBC and platelet therapy largely apply allogeneic cells (77% and 86%, respectively) in clinical settings. Still, the use of allogeneic RBCs requires blood type matching between donor and recipient. The major efforts in current RBC and platelet clinical trials are focused on optimizing transfusion protocols and verifying the durability of transfused cells.

Other than blood cells, stem cells account for 36% of current cell therapy trials (620/1705) as the second largest cell category of focus for this review. The trials of stem cell therapy, primarily those of HSCs and MSCs, encompass a wide range of indications covering 10 broad disease classifications (Figure 2). HSCs are multipotent stem cells capable of self‐renewing and differentiating into mature blood cells that form the myeloid and lymphoid cell lineages. As a result, hematopoietic stem cell transplantation (HSCT) can be used to reconstitute the hematopoietic and immunologic systems for the treatment of inherited and acquired blood disorders. HSCT is also used frequently to treat blood cancers after cancerous cells are eliminated by a myeloablative treatment.^29^ While autologous HSCs or matched sibling donor HSCs are the most ideal candidates for HSCT due to the reduced risk of GvHD, graft rejection, and engraftment syndrome,^30^ allogeneic HSCs have an advantage in cancer treatment because they can elicit graft‐versus‐tumor effects.^31^ MSCs, also a type of multipotent stem cell, are capable of effectively differentiating into a wide variety of cell types in mesodermal (e.g., chondrocytes), ectodermal (e.g., neurocytes), and endodermal lineages (e.g., hepatocytes).^32^ As a result, they have broad applications in clinical settings for the treatment of degenerative diseases, autoimmune diseases, inflammatory diseases, and trauma, among others. Notably, most stem cell therapy trials are in early stages with nearly equal representation in Phase 1 (44%) and Phase 2 (47%), showing their considerable potential to affect the future scope of cell therapies. Finally, microbes comprise 3% of the total trials (48/1705) with major indications including cancer (44%), infectious diseases (19%), and inflammatory diseases (13%). Although metabolic disorders account for only 8% of the indications for microbes, it is worth mentioning this unique niche, as very few cell therapies are investigated for this indication. Microbes exert therapeutic mechanisms of action by (i) displacing pathogenic microbiomes to restore symbiosis and (ii) producing therapeutic biomolecules, a function enabled by genetic modification.^33^

## 3 CLINICALLY APPROVED PRODUCTS

In the following sub‐sections, we provide additional details on approved cell therapy products that presently include T cells, stem cells, and DCs (Table 1). We also discuss closely related modalities, namely the applications of donor blood products and microbe‐based therapies in the clinic. Of note, many of these approved cell therapies are being developed and evaluated in current trials for additional indications, as summarized in Table S2.

**TABLE 1:** Clinically approved cell therapies, grouped by cell type

Name [Trade Name](Manufacturer)	Cell source	Approved indications	Approval year	Notes
T cell				
Tisagenlecleucel [Kymriah®] (Novartis)	Autologous	Relapsed or refractory (r/r) B‐cell precursor acute lymphoblasticleukemia (ALL); r/r large B‐cell lymphoma after two or more lines of systemic therapy including 1) diffuse large B‐cell lymphoma (DLBCL) not otherwise specified, 2) high‐grade B‐cell lymphoma, and 3) DLBCL arising from follicular lymphoma	2017 (USFDA), 2018 (EMA), 2018 (Health Canada), 2019 (JMHW), Australia, Israel, Switzerland	GM; CAR/CD19 (receptor/target)
Axicabtagene ciloleucel [Yescarta®] (Kite)	Autologous	Relapsed or refractory (r/r) large B‐cell lymphoma after two or more lines of systemic therapy including 1) diffuse large B‐cell lymphoma (DLBCL) not otherwise specified, 2) primary mediastinal large B‐cell lymphoma, 3) high‐grade B‐cell lymphoma, and 4) DLBCL arising from follicular lymphoma	2017 (USFDA), 2018 (EMA), 2019 (Health Canada)	GM; CAR/CD19 (receptor/target)
Brexucabtagene autoleucel [Tecartus™] (Kite)	Autologous	Relapsed or refractory mantle cell lymphoma	2020 (USFDA)	GM; CAR/CD19 (receptor/target)
N/A [ImmunCell‐LC®] (Green Cross Cell)	Autologous	Hepatocellular carcinoma, brain tumors, and pancreatic cancer	2007 (KFDA), 2018 (as Orphan Drug Designation by USFDA)	NGM; TAA (target)
Stem cell—Hematopoietic stem cells				
HPC, Cord Blood [Allocord] (SSM Cardinal Glennon Children's Medical Center)	Allogeneic	Disorders affecting the hematopoietic system that are inherited, acquired, or result from myeloablative treatment	2011 (USFDA)	NGM
HPC, Cord Blood [Clevecord™] (Cleveland Cord Blood Center)	Allogeneic	Disorders affecting the hematopoietic system that are inherited, acquired, or result from myeloablative treatment	2016 (USFDA)	NGM
HPC, Cord Blood [Ducord™] (Duke University School of Medicine)	Allogeneic	Disorders affecting the hematopoietic system that are inherited, acquired, or result from myeloablative treatment	2012 (USFDA)	NGM
HPC, Cord Blood [Hemacord™] (New York Blood Center)	Allogeneic	Disorders affecting the hematopoietic system that are inherited, acquired, or result from myeloablative treatment	2011 (USFDA)	NGM
HPC, Cord Blood [N/A] (Clinimmune Labs, University of Colorado Cord Blood Bank)	Allogeneic	Disorders affecting the hematopoietic system that are inherited, acquired, or result from myeloablative treatment	2012 (USFDA)	NGM
HPC, Cord Blood [N/A] (MD Anderson Cord Blood Bank)	Allogeneic	Disorders affecting the hematopoietic system that are inherited, acquired, or result from myeloablative treatment	2018 (USFDA)	NGM
HPC, Cord Blood [N/A] (LifeSouth Community Blood Centers)	Allogeneic	Disorders affecting the hematopoietic system that are inherited, acquired, or result from myeloablative treatment	2013 (USFDA)	NGM
HPC, Cord Blood [N/A] (Bloodworks)	Allogeneic	Disorders affecting the hematopoietic system that are inherited, acquired, or result from myeloablative treatment	2016 (USFDA)	NGM
Betibeglogene autotemcel [Zynteglo™] (bluebird bio)	Autologous	Transfusion‐dependent thalassemia	2019 (EMA)	GM
N/A [Strimvelis®] (GlaxoSmithKline)	Autologous	Adenosine deaminase‐severe combined immunodeficiency (ADA‐SCID)	2016 (EMA)	GM
Stem cell—Mesenchymal stem cells				
N/A [Cellgram™] (Pharmicell)	Autologous	Acute myocardial infarction	2011 (KFDA)	NGM; BM‐MSC (cell subtype)
N/A [Cartistem®] (Medipost)	Allogeneic	Repetitive and/or traumatic cartilage degeneration, including degenerative osteoarthritis without age limit	2012 (KFDA)	NGM
N/A [Queencell®] (Anterogen)	Autologous	Subcutaneous tissue defects	2010 (KFDA)	NGM; adipose‐derived MSC (cell subtype)
Darvadstrocel [Alofisel®] (TiGenix NV/Takeda)	Allogeneic	Complex perianal fistulas in Crohn's disease	2018 (EMA)	NGM; adipose‐derived MSC (cell subtype)
N/A [Cupistem®] (Anterogen)	Autologous	Crohn's fistula	2012 (KFDA)	NGM; adipose‐derived MSC (cell subtype)
Remestemcel‐L [Prochymal®] (Osiris Therapeutics/Mesoblast Limited)	Allogeneic	Steroid‐refractory acute GvHD (pediatric)	2012 (Health Canada)	NGM; BM‐MSC (cell subtype)
N/A [TEMCELL® HS Inj.] (JCR Pharmaceutics)	Allogeneic	Acute GvHD following hematopoietic stem cell transplant	2015 (Japan)	NGM; BM‐MSC (cell subtype)
Lenzumestrocel [NeuroNata‐R®] (Corestem)	Autologous	Amyotrophic lateral sclerosis	2014 (KFDA)	NGM
N/A [Stemirac] (Unique Access Medical)	Autologous	Spinal cord injury	2018 (JMHW, conditional approval)	NGM
N/A [Stempeucel®] (Stempeutics)	Allogeneic	Critical limb ischemia due to Buerger's disease	2017 (DCGI, limited marketing approval)	NGM
Stem cell—Limbal stem cell				
N/A [Holoclar®] (Chiesi)	Autologous	Limbal stem cell deficiency	2015 (European Commission)	NGM
Dendritic cell				
Sipuleucel‐T [Provenge®] (Dendreon Corporation)	Autologous	Asymptomatic or minimally symptomatic metastatic castrate‐resistant (hormone‐refractory) prostate cancer	2010 (USFDA), 2013 (EMA)	NGM
N/A [CreaVax‐RCC®] (JW CreaGene)	Autologous	Metastatic renal cell carcinoma (post‐nephrectomy)	2007 (KFDA)	NGM
N/A [APCeden®] (APAC Biotech)	Autologous	Prostate cancer, ovarian cancer, colorectal cancer, non‐small cell lung carcinoma	2017 (CDSCO)	NGM

Abbreviations: Indications: GvHD, Graft versus host disease. Agencies: CDSCO, Central Drugs Standard Control Organization (CDSCO), aka Indian FDA; EMA, European Medicines Agency; DCGI, Drug Controller General of India; KFDA, Korea Food and Drug Administration; JMHW, Japanese Ministry of Health and Welfare; USFDA, The United States Food and Drug Administration. Notes: BM, bone marrow; CAR, chimeric antigen receptor; GM, genetically modified; NGM, nongenetically modified; TAA, tumor‐associated antigen.

### 3.1 T cells

A total of four T‐cell products have been approved globally as of 2020, three by the FDA (USA) and one by the Korea Food & Drug Administration (KFDA) (Table 1). All FDA‐approved T‐cell products are for *CAR‐T therapy*, which is a form of immunotherapy that uses T cells genetically modified with a CAR to recognize and destroy cancer cells.^34^ The two essential components of a CAR include (i) an *extracellular target binding* domain used to identify surface antigens on cancer cells and (ii) an *intracellular signaling* portion comprised of costimulatory and activation domains that initiate processes including activation, clonal expansion, and cell killing.^35^ New functional domains are now being explored in both preclinical and clinical settings with the aim of providing safer and more effective CAR‐T therapies. Of note, all approved CAR‐T products are autologous and contain CARs targeting CD19, a biomarker that is selectively expressed on the surface of B cells. Accordingly, CAR‐T cells are indicated for relapsed or refractory (r/r) B‐cell malignancies.

Kymriah®, approved by the FDA in 2017, was the first T‐cell therapy available in the United States.^36^ Kymriah® is indicated for the treatment of children and young adults with r/r B‐cell precursor acute lymphoblastic leukemia (r/r B cell ALL) and adult patients with certain types of r/r large B‐cell lymphoma after the failure of at least two lines of systemic therapy. Yescarta® also received FDA approval in 2017 for the treatment of certain types of r/r large B‐cell lymphoma in adult patients who resist two or more lines of systemic therapy. Both Kymriah® and Yescarta® are being investigated in current clinical trials for additional liquid cancers (Table S2). Notable differences between Kymriah® and Yescarta® include their costimulatory domains (4‐1BB vs. CD28) and the associated persistence of the infused CAR‐T cells (1–7 years vs. <6 weeks).^37^, ^38^ In 2020, a third CAR‐T product, Tecartus™, received approval from the FDA to treat adults with r/r mantle cell lymphoma (MCL), which is an aggressive, rare form of non‐Hodgkin lymphoma. Unlike the two previously approved CAR‐T therapies, the Tecartus™ manufacturing process incorporates an additional step to enrich the T‐cell population and remove circulating tumor cells (CTCs) from patients' leukapheresis material. This process prevents CAR‐T cell activation and subsequent exhaustion during ex vivo manufacturing.^39^

Although CAR‐T products are indicated solely for hematological malignancies, other T cells have been used to treat solid tumors. ImmunCell‐LC®, an autologous cytokine‐induced killer (CIK) cell‐based immunotherapy, was approved by the KFDA in 2007 and earned orphan drug designation from the FDA in 2018. It is employed as an adjuvant therapy after tumor resection, and has been used for the treatment of hepatocellular carcinoma, brain tumors, and pancreatic cancer by eliminating residual tumor cells. ImmunCell‐LC® is manufactured by isolating PBMCs and incubating them with interleukin‐2 (IL‐2) and anti‐CD3 antibody,^40^ to collect activated T lymphocytes. ImmunCell‐LC® showed an increased rate of recurrence‐free and overall survival in patients who underwent tumor resection.^41^ Additional clinical trials of ImmunCell‐LC® are underway for hepatocellular carcinoma.

### 3.2 Stem cells

Our search revealed a total of 21 stem cell products that have been approved globally, with 12 approved by the FDA (USA) or European Medicines Agency (EMA, Europe). The remaining nine products are approved in other countries, particularly in Asia (Table 1). Notably, all but one product are composed of HSCs or MSCs.

#### 3.2.1 Hematopoietic stem cells

There are 10 approved HSC products globally, with eight approved by the FDA and the remaining two by the EMA (Table 1). The FDA approved the first batch of products, Allocord and Hemacord™, in 2011. Subsequently, six more similar products were FDA approved, with the most recent in 2018. All of these products are cord blood‐based therapies that have applications for malignant and nonmalignant blood disorders and immunodeficiency disorders. Notably, cord blood‐based HSCs offer considerable advantages over other forms of allogeneic HSCT, such as easier accessibility, higher tolerance for human leukocyte antigen (HLA) mismatch, and a lower risk of GvHD.^42^ While pediatric HSCT is still exclusively performed with HLA‐matched cord blood from a sibling,^43^ the tolerance for 1–2 HLA‐A, ‐B and ‐DR mismatches has loosened to enable the considerable expansion of the HSCT‐eligible adult patient population.

In addition, Strimvelis® and Zynteglo™ are autologous HSC‐based gene therapies that have been EMA approved. Strimvelis®, EMA approved in 2016, is indicated for adenosine deaminase deficiency (ADA‐SCID), an immunodeficiency disorder caused by mutations in the gene coding for adenosine deaminase (ADA). Zynteglo™, EMA approved in 2019, is employed for transfusion‐dependent thalassemia, a genetic disorder caused by mutations in the β‐globin gene that result in considerably reduced or absent adult hemoglobin. Zynteglo™ uses the lentiviral vector LentiGlobin BB305 to transduce autologous CD34+ cells with the β‐globin gene. These cells are then infused back to the patient and traffic to the bone marrow, where they differentiate into mature RBCs with functional hemoglobin.^44^ Several current clinical trials are exploring the use of LentiGlobin BB305 for applications including thalassemia and sickle cell disease.

#### 3.2.2 Mesenchymal stem cells

There are 10 MSC products that have been approved globally as of 2020 (Table 1), although none have been approved by the FDA. The current MSC products fall into two major categories according to their mechanisms of action and approved indications: (i) tissue repair and (ii) immunomodulation.

MSCs have multipotent potential and can differentiate into a variety of cell types, such as osteoblasts, chondrocytes, myocytes, adipocytes, and neuronal cells.^45^, ^46^ Based on this biological function, three MSC therapies have been approved for tissue repair applications (Table 1). Cellgram™, an autologous MSC therapy, was approved by the KFDA in 2011 for acute myocardial infarction. Mechanisms of action of Cellgram™ are reported to involve (i) MSCs' capability to differentiate into cardiac myocytes and (ii) MSCs' pleiotropic secretomes that promote angiogenesis.^47^ Another MSC‐based tissue repair product, Cartistem®, was approved by the KFDA in 2012 for repetitive and/or traumatic cartilage degeneration, including degenerative osteoarthritis.^48^ Queencell®, an autologous adipose‐derived cell product, was also approved by the KFDA, in 2010, for the treatment of subcutaneous tissue defects. However, unlike other approved MSCs, Queencell® is not composed of pure MSCs and is instead comprised of a mixture of MSCs, pericytes, mast cells, fibroblasts, and endothelial progenitor cells.

MSCs also have immunomodulatory capabilities that can be used to regulate immune responses in many pathologies. Based on this capability, seven MSC products have been approved for indications including Crohn's fistula (Alofisel®, Cupistem®), acute GvHD (aGvHD) (Prochymal®, TEMCELL®), amyotrophic lateral sclerosis (ALS) (NeuroNata‐R®), spinal cord injury (Stemirac), and critical limb ischemia due to Buerger's disease (Stempeucel®) (Table 1). Alofisel®, an allogeneic MSC therapy for complex perianal fistula in Crohn's disease, is the only MSC product approved by the EMA. Its mechanism of action seems to involve MSCs' ability to inhibit the proliferation of activated lymphocytes and thereby reduce pro‐inflammatory cytokine production.^49^ A similar product, Cupistem®, is an autologous adipose‐derived MSC product that received approval from the KFDA in 2012 to treat patients with Crohn's fistula. In 2012, Prochymal® received approval from the Canadian Food Inspection Agency (CFIA) for the treatment of steroid‐refractory acute GvHD (SR‐aGvHD) in pediatric patients. Of note, Prochymal® showed evidence of safety, tolerability, and efficacy as a first‐line therapy after initial steroid failure in pediatric patients with SR‐aGvHD in a Phase 3 trial.^50^ However, the FDA denied its approval this year and recommended at least one more randomized controlled trial in adults and/or children to provide additional information about the therapeutic mechanism and efficacy.

A few of these MSC products (e.g., Cellgram™, Cartistem®, Prochymal®) are being evaluated in current clinical trials for additional indications including alcoholic liver cirrhosis, acute respiratory distress syndrome (ARDS) due to coronavirus disease 2019 (COVID‐19), and osteochondral lesions (Table S2). In addition to the approved HSC and MSC products, Holoclar®, an autologous LSC product, won EMA approval in 2015 for the treatment of LSC deficiency secondary to ocular burns. However, since Holoclar® is given to patients in the form of a cornea sheet rather than a single‐cell suspension, it is not within the scope of this review.

### 3.3 Dendritic cells

There are currently three DC products in the global market with approvals by the FDA, KFDA, and Indian FDA (Table 1). Provenge® won FDA approval in 2010 for the treatment of metastatic castrate‐resistant prostate cancer. Of note, it is the first cell therapy used as a cancer vaccine in the United States.^51^ To produce Provenge®, the patient's leukocytes are collected and then expanded ex vivo with a prostate cancer tissue antigen (prostatic acid phosphatase [PAP]) and granulocyte‐macrophage colony‐stimulating factor (GM‐CSF). This autologous multicell suspension composed primarily of DCs, but also other leukocytes, is administered intravenously in three doses, each separated by 2 weeks. The main mechanism of action is the DC‐mediated presentation of PAP to the patient's T cells, which elicits an adaptive immune response against the prostate cancer cells. While Provenge® is the only DC therapy approved by the FDA, CreaVax®, an autologous DC therapy, was approved by the KFDA in 2007 for renal cell carcinoma. Similarly, APCeden® is an autologous DC therapy approved by the Indian FDA in 2017 for the treatment of prostate, ovarian, colorectal, and non‐small cell lung cancers.

### 3.4 Other cell‐based therapies (transfusions, transplants, and supplements)

While donor blood products have a long history in the treatment of some blood disorders and deficiencies,^15^ there are no specific approved products for RBCs and platelets. RBCs are administered to patients who are anemic due to a blood disorder (i.e., thalassemia, sickle cell disease, iron or other vitamin deficiency, aplastic anemia), or as a result of trauma or injury. Prior to intravenous administration, blood must be ABO blood type and Rhesus D (RhD) matched. Packed RBC infusions are given most commonly, although whole blood can also be administered. In many cases, autologous blood is isolated prior to a surgical procedure in anticipation of potential blood loss. Currently, drugs cannot be mixed with donor blood prior to infusion. Platelet transfusions are indicated for the treatment of thrombocytopenia, which can occur as a result of disease or in response to cancer treatment. Current clinical studies continue to investigate the range of suitable storage conditions and dosing regimens for donor platelets.

To the best of our knowledge, there are no microbe‐based therapies that have been approved for clinical use by the FDA or EMA. However, there are two closely related modalities currently used in the clinic. The first are fecal microbiota transplants (FMTs), in which a solution of fecal matter from a healthy donor is supplied to the intestinal tract of the patient to alter the gut microbiome composition.^52^ While these transplants are used to treat a variety of diseases in clinical settings, they are beyond the scope of this review because they are not being developed as individual drug products.^53^, ^54^, ^55^ The second type of therapy, probiotics, includes living microorganisms that are widely available over the counter and can also be prescribed by clinicians.^56^, ^57^, ^58^ However, they are also beyond the scope of this review because they are typically categorized as foods, functional foods, or supplements, and as such do not undergo the same regulatory process as pharmaceuticals.^59^

## 4 CURRENT CLINICAL TRIALS

In the following sub‐sections, we categorize and discuss current clinical trials employing blood cells and stem cells administered as single‐cell suspensions, and on microbes administered in various dosage forms. We define current clinical trials as those that appear on clinicaltrials.gov with a status of not yet recruiting, recruiting, enrolling by invitation, or active/not recruiting. These sub‐sections account for data that capture the current clinical landscape as of August 2020. The overall summary of our analysis and additional details are shown in Figure 2.

### 4.1 T cells

The clinical landscape of T‐cell therapies has rapidly diversified over the past 10 years. Today, T cells are the most investigated cell type among all cell therapy trials, encompassing 45% of the total trials (Figure 2 **)**. A representative selection of T‐cell trials is shown in Table 2. In our analysis, we grouped 767 T‐cell trials into four main categories. We first classified them according to genetic modification status as genetically modified (GM) or nongenetically modified (NGM). Cells in the GM category were then classified according to receptor type, either CAR or T‐cell receptor (TCR). Cells in the NGM category were classified according to the type of target, as these cells are usually trained ex vivo to target either tumor‐associated antigens (TAA) or viral antigens (virus‐specific, VST) via endogenous TCRs. Trials that could not be sorted into one of these categories were labeled “not applicable (N/A)” if there was no stated receptor or target, or “Other” if there were unique features that prevented clear classification, such as receptors that could not be readily identified as a CAR or TCR.

**TABLE 2:** Examples of current clinical trials for T‐cell therapies, grouped by indication and cell type

Cell type	GM	Receptor type	Target	Source	Indication	Name (Sponsor)	Route	Payload	Trial number	Other trials and/or notes
Cancer (*n* = 683)										
T cell	No	TAA	NY‐ESO‐1, MAGE‐A4, PRAME, survivin, and/or SSX	Autologous	Lymphoma	N/A (Baylor College of Medicine)	IV	N/A	NCT01333046 (Phase 1)	NCT03192462 (Phase 1/2), NCT02239861 (Phase 1), NCT03093350 (Phase 2)
T cell	No	Virus specific	EBV	Autologous	Nasopharyngeal carcinoma	N/A (Tessa Therapeutics)	IV	N/A	NCT02578641 (Phase 3)	None
T cell	Yes	CAR	CEA	Autologous	Liver metastasis	Anti‐CEA CAR‐T (Sorrento Therapeutics)	Hepatic artery	N/A	NCT04037241 (Phase 2/3)	NCT03818165 (Phase 1), NCT02850536 (Phase 1), NCT03682744 (Phase 1)
T cell	Yes	CAR	CD19, CD20	Autologous	Leukemia, lymphoma	CD20‐CD19 cCAR (iCell Gene Therapeutics)	IV	N/A	NCT04156178 (Phase 1)	Dual CAR‐T
T cell	Yes	CAR	BCMA	Autologous	Multiple myeloma	bb2121 (Celgene)	IV	N/A	NCT03651128 (Phase 3)	NCT03361748 (Phase 2), NCT03601078 (Phase 2), NCT02658929 (Phase 1), NCT04196491 (Phase 1)
T cell	Yes	CAR	CD19	Allogeneic	Lymphoma	CTX 110 (CRISPR Therapeutics)	IV	N/A	NCT04035434 (Phase 1/2)	CRISPR/Cas9 edited
T cell	Yes	TCR	MAGE‐A10	Autologous	Urothelial carcinoma	MAGE‐A10c796T (Adaptimmune)	IV	N/A	NCT02989064 (Phase 1)	NCT02592577 (Phase 1)
T cell	Yes	TCR	HPV	Autologous	Oropharyngeal cancer	E7 TCR T (National Cancer Institute)	IT	N/A	NCT04044950 (Phase 2)	NCT04476251 (Early Phase 1), NCT02858310 (Phase 1/2), NCT04015336 (Phase 2)
T cell	Yes	CAR	CD19	Autologous	Leukemia, lymphoma	N/A (Uppsala University)	IV	N/A	NCT03068416 (Phase 2)	Third‐generation CAR
T cell	Yes	CAR	GPC3	Autologous	Solid tumors	AGAR T Cells (Baylor College of Medicine)	IV	iCas9 suicide gene, IL‐15 secretion	NCT04377932 (Phase 1)	None
T cell	Yes	TCR	HA‐1	Allogeneic	Leukemia	N/A (Fred Hutchinson Cancer Center)	IV	Suicide gene	NCT03326921 (Phase 1)	None
T cell (TIL)	No	N/A	N/A	Autologous	Renal cell carcinoma	Nivo‐TIL (Nantes University Hospital, Bristol Meyers Squibb)	IV	N/A	NCT03374839 (Phase 1)	None
T cell	No	TAA	WT1	Allogeneic	Leukemia	WT1‐CTL (Atara Biotherapeutics)	IV	N/A	NCT00620633 (Phase 1)	None
T cell	Yes	CAR	IL13Rα2	Autologous	Glioblastoma	N/A (City of Hope Medical Center)	IT, IC, IVN	CD19t suicide gene	NCT02208362 (Phase 1)	NCT04510051 (Phase 1), NCT04003649 (Phase 1) Second‐generation CAR
T cell	Yes	TCR	CD19	Autologous	Lymphoma	ET1901 ARTEMIS™ (Eureka Therapeutics)	IV	N/A	NCT03415399 (Phase 1)	Novel TCR
Transplant‐related diseases (*n* = 63)										
T cell	No	Virus‐specific	CMV, EBV, ADV	Allogeneic	Viral infection	N/A (University of Pittsburgh)	IV	N/A	NCT04364178 (Phase 1/2)	None
T cell (Treg)	No	N/A	N/A	Allogeneic	GvHD	TREG2015001 (European Commission)	IV	N/A	NCT02749084 (Phase 1/2)	None
T cell	No	TCR	EBV	Allogeneic	EBV‐associated post‐transplant lymphoproliferative disease	Tabelecleucel (Atara Biotherapeutics)	IV	N/A	NCT03392142 (Phase 3)	NCT03769467 (Phase 1/2), NCT03394365 (Phase 3)
Infectious diseases (*n* = 24)										
T cell	No	Virus‐specific	HIV	Autologous	Viral infection	HXTC (University of North Carolina, Chapel Hill)	IV	N/A	NCT03212989 (Phase 1)	None
T cell	Yes	CAR	HIV	Autologous	Viral infection	N/A (Guangzhou 8th People's Hospital)	IV	N/A	NCT03240328 (Phase 1)	NCT03980691 (Phase 1)
T cell	No	Virus‐specific	SARS‐CoV‐2	Allogeneic	Viral infection	N/A (Children's Hospital Medical Center, Cincinnati)	IV	N/A	NCT04406064 (Phase 1)	None
Autoimmune diseases (*n* = 12)										
T cell (Treg)	No	N/A	N/A	Allogeneic	Type I diabetes	N/A (Second Xiangya Hospital of Central South University)	IV	N/A	NCT03011021 (Phase 1/2)	NCT02932826 (Phase 1/2) Cord blood‐derived
Non‐autoimmune inflammatory diseases (*n* = 3)										
T cell (Treg)	No	N/A	N/A	Autologous	Crohn's disease	TR004 (King's College London)	IV	N/A	NCT03185000 (Phase 1/2)	None
Other (*n* = 19)										
T cell	Yes	CAR	BCMA	Autologous	Myasthenia gravis	Descartes‐08 (Cartesian Therapeutics)	IV	N/A	NCT04146051 (Phase 1/2)	NCT03448978 (Phase 1/2)
T cell (Treg, Th2)	No	N/A	N/A	Autologous	Amyotrophic lateral sclerosis	RAPA‐501 (Rapa Therapeutics)	IV	N/A	NCT04220190 (Phase 1/2)	Rapamycin resistant

Abbreviations: Cell Subtypes: CAR, chimeric antigen receptor; TAA, tumor‐associated antigen; TCR, T‐cell receptor; TIL, tumor‐infiltrating lymphocyte; Treg, regulatory T cell; Th2, T helper cell type 2. Targets: ADA, adenosine deaminase; ADV, adenovirus; BCMA, B‐cell maturation antigen; Bcl11a, B‐cell leukemia/lymphoma 11a; CMV, cytomegalovirus; CD, cluster of differentiation; CEA, carcinoembryonic antigen; CCR5, C‐C chemokine receptor type 5; EBV, Epstein Barr virus; GPC3, glypican 3; HER2, human epidermal growth factor receptor 2; HA‐1, minor histocompatibility antigen; HPV, human papillomavirus; HIV, human immunodeficiency virus; IL13Ra2, IL‐13 receptor alpha 2; MAGEA4, melanoma‐associated antigen 4; MAGE‐A10, melanoma‐associated antigen 10; NY‐ESO‐1, New York esophageal squamous cell carcinoma‐1; NKG2DL, natural killer group 2D ligand; PRAME, preferentially expressed antigen in melanoma; Rev, regulator of expression of virion proteins; SSX, synovial sarcoma X chromosome breakpoint; SARS‐CoV‐2, severe acute respiratory syndrome coronavirus 2; Tat, transactivator of expression; WT1, Wilms tumor antigen 1. Indications: GvHD, graft versus host disease. Routes: IV, intravenous; IT, intratumoral; IC, intracavitary; IVN, intraventricular. Payloads: iCas9, inducible caspase 9; CD19t, truncated CD19.

**FIGURE 3:**
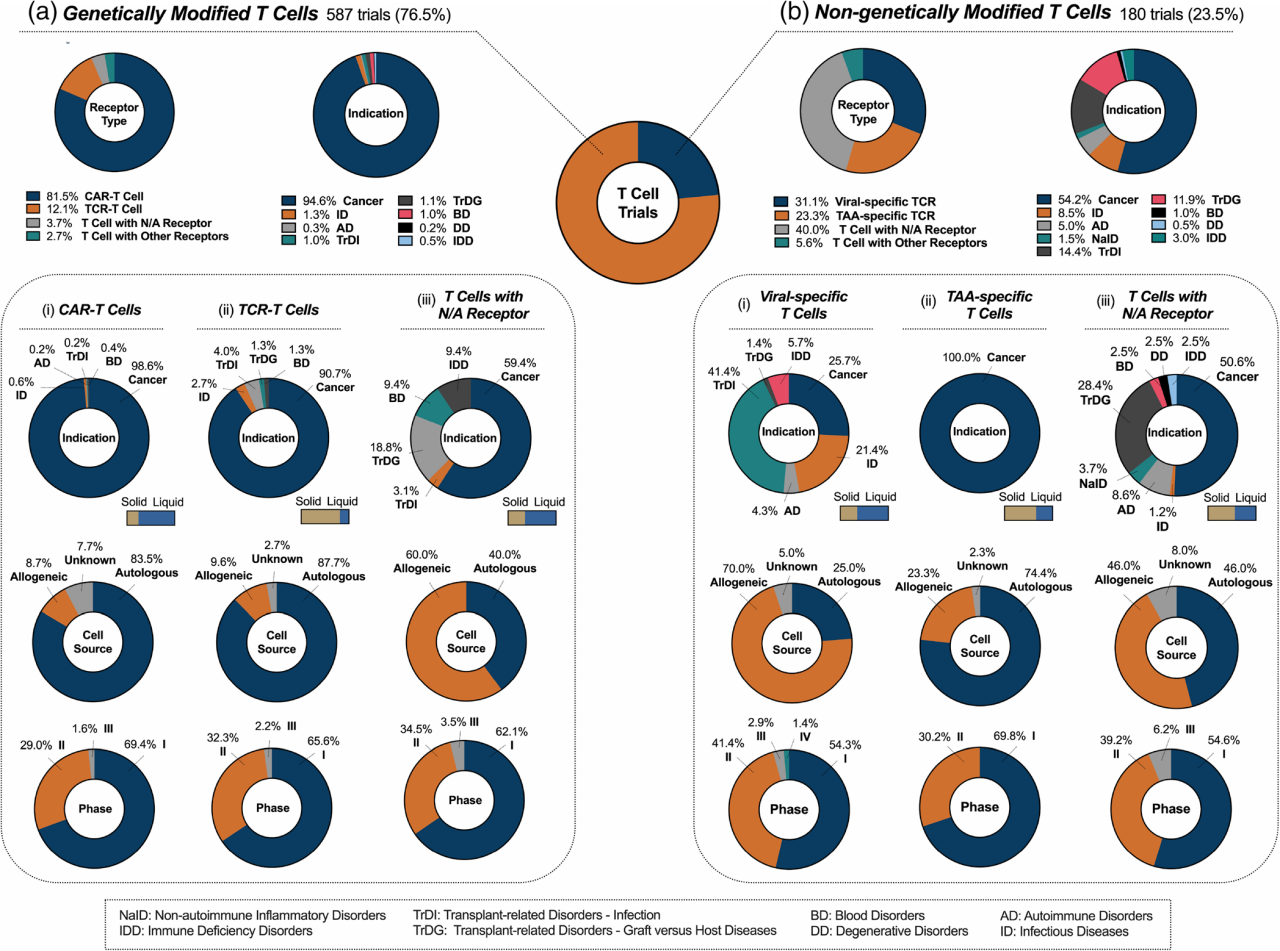
Current landscape of T‐cell clinical trials; 767 T cell clinical trials were analyzed and classified according to genetic modification status as: (a) genetically modified (GM) or (b) nongenetically modified (NGM) T cells. Trials in the GM category were further classified according to the type of genetically modified receptors: (i) CAR‐T cells, (ii) TCR‐T cells, or (iii) T cells with receptor that is not applicable (N/A). Similarly, trials in the NGM category were further classified according to the type of target: (i) virus‐specific T cells, (ii) TAA‐specific T cells, or (iii) T cells with receptor that is not applicable (N/A)

About 77% of the T‐cell clinical trials involve GM cells; this is expected given the dominance of CAR‐T therapy in the field (Figure 3). Indeed, 82% of the trials involving GM cells, or 63% of all T‐cell trials, include T cells with a CAR modification **(**Figure 3(a) **)**. However, this subset is highly diverse. While all three approved CAR‐T therapies include a single anti‐CD19 CAR for the treatment of B‐cell malignancies, CD19 comprises only 37% of all targets in the clinical landscape (Table S3). The next most abundant targets include BCMA, CD22, and CD20, all of which are also exclusively found on B cells (Table S3). Together with CD19, they comprise 58% of the targets that are currently investigated in CAR‐T clinical trials, reflecting the continued prevalence of B‐cell cancer indications in the field. One new approach for the treatment of liquid cancers is dual CAR‐T therapy, in which two different CARs are presented on the same cell or two distinct CAR‐T products are co‐infused.^60^ This strategy has the potential to reduce relapse rates by targeting and eliminating cancer cells that are resistant to CD19‐targeted therapy (NCT04049383).

Historically, CAR‐T therapy been ineffective in the treatment of solid tumors due to a lack of defined extracellular antigen targets, insufficient T‐cell infiltration, and challenges overcoming immunosuppressive tumor microenvironments.^61^, ^62^ Still, about 24% of CAR‐T targets in the clinical landscape are found on solid tumors (Figure 3(a) (i) bar) with the most common targets being GD2 (neuroblastomas and melanomas), mesothelin (mesothelioma and cancers of the ovary, pancreas, colon, etc.), HER2 (metastatic breast cancers), and GPC3 (liver cancers) (Table S3). Based on our analysis, intravenous administration remains the most common route of administration for CAR‐T therapy against solid tumors (75%), though other administration routes are also being investigated, such as intraperitoneal, intraventricular/intracavitary, intratumoral, intra‐hepatic artery, intrapancreatic (via splenic vein or artery), and intrapleural administration. The emergence of strategies to prevent T‐cell exhaustion, improve target specificity, and promote tissue infiltration may expand the current application of CAR‐T therapies against solid tumors (Section 5).^62^

The next major class of GM T cell is the TCR‐T cell, which comprises about 12% of trials involving GM T cells **(**Figure 3(a) **)**. While most current CARs are only capable of recognizing cell surface antigens, TCRs recognize peptides derived from intracellular proteins by binding to major histocompatibility complex (MHC) class I molecules on target cells. As a result, 81% of TCR‐T trials have targets that are exclusive to solid tumors (Figure 3(a) (ii) bar and Table S4), as these cancers often do not overexpress a readily identifiable extracellular antigen that can be recognized by a CAR. Common indications include hepatocellular carcinoma, melanoma, and head and neck squamous cell carcinoma. TCR‐T cells are mostly autologous (88%) (Figure 3(a) (ii)) with a small yet burgeoning group of allogeneic therapies, most of which are specific for Epstein–Barr virus (EBV). The TCR category also includes some novel, TCR‐like receptors with unique properties. For example, TCRs containing an antigen‐binding fragment (Fab) coupled with portions of native TCR chains can expand TCR targeting ability to extracellular antigens and has also shown reduced cytotoxicity compared to other engineered TCRs (NCT03415399).^61^, ^63^

There also exists a subset of GM T cells that are not transduced with a receptor (Figure 3(a) (iii)), which, unlike CAR‐ and TCR‐T cells, predominantly use allogeneic cells. This subset most often contains genetic modifications that enable the display of an inducible suicide gene (discussed in Section 5), expression of receptors, or secretion of therapeutic payloads like cytokines. The T‐cell subtypes in this group are diverse and include regulatory T cells (Tregs), tumor‐infiltrating lymphocytes (TILs), and helper T cells in addition to general peripheral populations. For example, BPX‐501 is an allogeneic, suicide gene‐transduced polyclonal T cell replacement product designed to prevent GvHD and improve immune reconstitution after HSCT (NCT02065869, NCT02231710). The small subset of GM cell trials classified as “Other” either contain synthetic receptors that cannot be classified as a CAR or TCR, or combinations of more than one receptor type. Many of these include CAR‐T cells that also have endogenous TCRs for viral‐specific targets, enabling the dual targeting of cancers induced by oncogenic viruses.

NGM T cells account for a 24% of the total T‐cell trials (Figure 3(b)), yet they have broader indications compared to GM T cells. T cells that have been primed to target specific antigen(s) via endogenously expressed TCRs can be broadly classified into viral‐specific T (VST) and TAA‐specific T (TAA‐T) cells. VSTs are being clinically evaluated for treatment of cancer, transplant‐related diseases, and infection. They can be used to eliminate virus‐induced cancers by recognizing viral epitopes presented on MHC class I molecules of infected cells (NCT02578641). Another subset of VSTs are designed to treat viral infections occurring after a HSCT (NCT04364178). In this case, VSTs can be used prophylactically or following diagnosis to prevent infection‐associated complications and improve transplant engraftment.^64^ The most common targets for this application are cytomegalovirus (CMV) and EBV (Table S5). Finally, VSTs may also be used to treat viral infections that are not related to cancer and/or transplant, such as HIV (NCT03212989) and severe acute respiratory syndrome coronavirus 2 (SARS‐CoV‐2) (NCT04406064). VSTs are usually derived from an allogeneic source (70%) **(**Figure 3(b) (i)), as they are often isolated from transplant donors to ensure compatibility with HSCT recipients, or from convalescent donors for the treatment of viral infections. Some VSTs have already progressed to clinical trials in Phases 3 and 4.

Unlike VSTs, TAA‐Ts are mostly autologous (74%) (Figure 3(b) (ii)). NY‐ESO‐1, PRAME, and survivin are the most commonly named targets; however, we should note that the majority of the targets were not specified in the trial listings. That is, many trials indicated that the cells could be personalized for each patient, with the therapy consisting of a mixture of cells designed to target multiple TAAs. This explains the benefit of autologous sourcing, as these mixed populations of activated T cells can be isolated directly from the patient. About two thirds of these therapies are designed to treat solid tumors **(**Figure 3(b) (ii) bar**)**, arising from the ability of endogenous TCRs to recognize intracellular antigens via display on MHC molecules. There are multiple trials currently in Phase 2 (NCT03093350), although TAA‐Ts have yet to progress to Phase 3.

The remaining NGM cell trials with N/A receptors (40% of all NGM) comprise a broad spectrum of T‐cell subtypes and use T cells that do not have a specific antigen target (Figure 3(b) (iii)). These untargeted NGM cells are often derived from a general, unsorted population of peripheral T cells. Of these trials, a total of six have progressed to Phase 2/3 or Phase 3 (NCT03944980, NCT02999854). Other therapies in earlier clinical stages (Phases 1 and 2) consist of more specific populations. For example, TILs are being explored as a NGM solid tumor‐targeting therapy (NCT03374839). Tregs, on the other hand, are primarily being used for the treatment of GvHD and autoimmune disorders. Based on our analysis, they comprise a large portion of the NGM cells without specific targets (28/72, 39%) and contribute mainly to the transplant‐related disorders—graft versus host diseases (TrDG) indication (NCT01903473, NCT02749084). Tregs promote immune tolerance in both antigen‐dependent and independent manners, including the secretion of anti‐inflammatory cytokines to dampen immune‐mediated tissue damage.^65^

### 4.2 Stem cells

**FIGURE 4:**
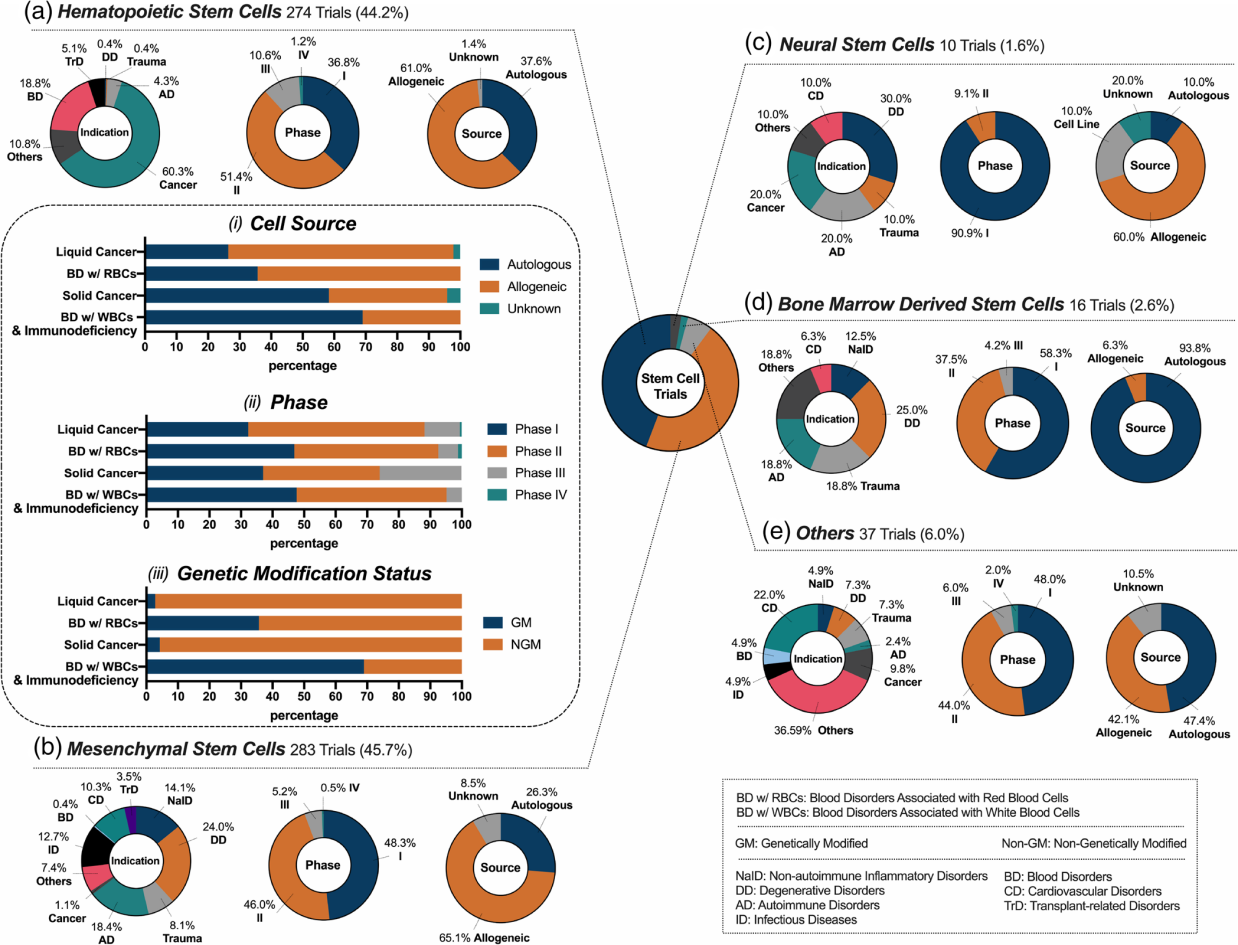
Current landscape of stem cell clinical trials; 620 stem cell clinical trials were analyzed and classified as one of the following types: (a) hematopoietic stem cells, (b) mesenchymal stem cells, (c) neural stem cells, (d) bone marrow‐derived stem cells and (e) others (cardiac stem cells, limbal stem cells, and endothelial progenitor cells)

Stem cells have persisted at the forefront of the clinical landscape for cell therapies due to their high multipotency, which enables their differentiation into various types of mature cells with a broad range of functions. Currently, stem cells account for 36% of total trials with applications covering 10 major indications (Figure 2). A representative selection of these trials is provided in Table 3. In our analysis, we first categorized stem cell trials by cell subtype, including HSCs (44%), MSCs (46%), neural stem cells (NSCs, 2%), bone marrow‐derived stem cells (BMDSCs, 3%), and others (Figure 4). Cells in the HSC and MSC categories were further classified according to indications and features like genetic modification.

**TABLE 3:** Examples of current clinical trials for stem cell therapies, grouped by indication and cell type

GM	Sub‐type	Gene of interest	Source	Indication	Name (Sponsor)	Route	Trial number	Other trials and/or notes
Degenerative diseases (*n* = 79)								
No	MSC	N/A	Autologous	Osteoarthritis	JOINTSTEM® (Nature Cell Co. Ltd.)	IA	NCT03990805 (Phase 3)	NCT03509025 (Phase 2/3), NCT04427930 (Phase 3), NCT04368806 (Phase 2/3)
No	MSC	N/A	Autologous	Alzheimer's disease	HB‐adMSCs (Hope Biosciences)	IV	NCT04228666 (Phase 1/2)	None
No	MSC	N/A	Allogeneic	Parkinson's disease	N/A (The University of Texas Health Science Center, Houston)	IV	NCT04506073 (Phase 2)	None
No	MSC	N/A	Autologous	Huntington's disease	Cellavita‐HD (Azidus Brasil)	IV	NCT03252535 (Phase 2)	NCT04219241 (Phase 2/3), NCT02728115 (Phase 1)
No	NSC	N/A	Allogeneic	Parkinson's disease	ISC‐hpNSC (Cyto Therapeutics Pty Limited)	ICe	NCT02452723 (Phase 1)	None
Autoimmune diseases (*n* = 70)								
No	MSC	N/A	Allogeneic	Rheumatoid arthritis	FURESTEM‐RA Inj. (Kang Stem Biotech Co., Ltd.)	IV	NCT03618784 (Phase 1/2)	None
No	MSC	N/A	Allogeneic	Type 1 diabetes	N/A (Medical University of South Carolina)	IV	NCT04061746 (Phase 1)	None
No	MSC	N/A	Autologous	Multiple sclerosis	NurOwn® (MSC‐NTF) (Brainstorm‐Cell Therapeutics)	IV	NCT03799718 (Phase 2)	Neurotrophic factor secretion
No	MSC	N/A	Allogeneic	Systemic lupus erythematosus	N/A (Medical University of South Carolina)	IV	NCT02633163 (Phase 2)	None
No	NSC	N/A	Allogeneic	Multiple sclerosis	hNSCs (IRCCS San Raffaele)	ITh	NCT03269071 (Phase 1)	None
Cancer (*n* = 176)								
No	MSC	N/A	Allogeneic	Ovarian cancer	N/A (Mayo Clinic)	IP	NCT02068794 (Phase 1/2)	Oncolytic measles virus encoding thyroidal sodium iodide symporter as payload
No	MSC	N/A	Allogeneic	Non‐small cell lung cancer	MSCTRAIL (University College London)	IV	NCT03298763 (Phase 1/2)	TRAIL as payload
Yes	HSC	shI; TAR; CCR5RZ	Unknown	AIDS‐related liquid cancers	N/A (City of Hope Medical Center)	IV	NCT02337985 (Phase 1)	NCT01961063 (Phase 1); HSC transfected by lentiviral vector rHIV7‐shI‐TAR‐CCR5RZ to express i) a short hairpin RNA (shRNA) targeted to an exon of the HIV‐1 genes tat/rev(shI), (ii) a decoy for the HIV TAT reactive element (TAR), and (iii) a ribozyme targeting the host cells CCR5 chemokine receptor (CCR5RZ)
No	HSC	N/A	Autologous	Non‐Hodgkin lymphoma	N/A (Novartis Pharmaceuticals)	IV	NCT03570892 (Phase 2/3)	None
No	HSC	N/A	Allogeneic	Pediatric AML	N/A (Samsung Medical Center)	IV	NCT02848183 (Phase 2)	None
No	HSC	N/A	Autologous	Brain and CNS tumors	N/A (St. Jude Children's Research Hospital)	IV	NCT00085202 (Phase 3)	None
No	HSC	N/A	Autologous	Germ cell tumors	N/A (Masonic Cancer Center, University of Minnesota)	IV	NCT00432094 (Phase 2)	None
No	HSC	N/A	Allogeneic	Solid tumors	N/A (M.D. Anderson Cancer Center)	IV	NCT04530487 (Phase 2)	None
Blood disorders (*n* = 55)								
No	HSC	N/A	Allogeneic	Thalassemia major	N/A (First Affiliated Hospital of Guangxi Medical University)	IV	NCT04009525 (Phase 4)	None
Yes	HSC	β‐globin	Allogeneic	Sickle cell disease	N/A (bluebird bio)	IV	NCT04293185 (Phase 3)	NCT02906202 (Phase 3 for thalassemia); Lentiviral vector LentiGlobin BB305 encoding functional β‐globin
Yes	HSC	Bcl11a	Autologous	Sickle cell disease	CTX001 (Vertex Pharmaceuticals Incorporated)	IV	NCT03745287 (Phase 1/2)	NCT03655678 (Phase 1/2); CRISPR/Cas9 editing to knock out enhancer region of Bcl11a
Transplant‐related diseases (*n* = 24)								
No	HSC	N/A	Allogeneic	Kidney transplant rejection	MDR‐102 (Medeor Therapeutics, Inc.)	IV	NCT03605654 (Phase 2/3)	None
No	MSC	N/A	Allogeneic	Steroid‐resistant severe aGvHD	N/A (Fujian Medical University)	IV	NCT03631589 (Phase 2/3)	None
Nonautoimmune inflammatory diseases (*n* = 44)								
No	MSC	N/A	Allogeneic	Non‐COVID‐19 ARDS	HC016 (Histocell, S.L.)	IV	NCT04289194 (Phase 1/2)	None
No	MSC	N/A	Allogeneic	COVID‐19	Remestemcel‐L (Mesoblast, Inc.)	IV	NCT04371393 (Phase 3)	None
No	MSC	N/A	Autologous	COPD	N/A (Mayo Clinic)	IV	NCT04047810 (Phase 1)	None
No	MAPC	N/A	Allogeneic	Non‐COVID‐19 ARDS	HLCM051 (Healios K.K.)	IV	NCT03807804 (Phase 2)	None
Trauma (*n* = 31)								
No	MSC	N/A	Autologous	Spinal cord injury	N/A (Mayo Clinic)	ITh	NCT04520373 (Phase 2)	None
No	MSC	N/A	Allogeneic	Ischemia reperfusion Injury	N/A (Mayo Clinic)	IAe	NCT04388761 (Phase 2)	None
No	MSC	N/A	Autologous	Traumatic brain injury	HB‐adMSCs (Hope Biosciences)	IV	NCT04063215 (Phase 1/2)	None
No	MSC	N/A	Allogeneic	Heart failure	N/A (MD Anderson Cancer Center)	IV	NCT02408432 (Phase 1)	None
No	MSC	N/A	Allogeneic	Acute‐on‐chronic liver failure	allo‐APZ2‐ACLF (RHEACELL GmbH & Co. KG)	IV	NCT03860155 (Phase 1/2)	None
Other (*n* = 70)								
Yes	HSC	CCR5	Autologous	DOCK8 deficiency	SB‐728mR‐HSPC (City of Hope Medical Center)	IV	NCT02500849 (Phase 2)	CCR5 modified by zinc finger nuclease
Yes	HSC	ADA	Autologous	SCID due to ADA deficiency	OTL‐101 (Great Ormond Street Hospital for Children NHS Foundation Trust)	IV	NCT03765632 (Phase 1/2)	TYF‐ADA lentiviral vector encoding functional ADA gene
Yes	HSC	Human adrenoleukodystrophy protein	Autologous	Cerebral adrenoleukodystrophy	Elivaldogene autotemcel (bluebird bio)	IV	NCT03852498 (Phase 3)	NCT01896102 (Phase 2/3); Lenti‐D lentiviral vector encoding cDNA for human adrenoleukodystrophy protein
Yes	HSC	CD18	Autologous	Leukocyte adhesion deficiency	RP‐L201 (Rocket Pharmaceuticals)	IV	NCT03812263 (Phase 1/2)	NCT03825783 (Phase 1); Delivery of lentiviral vector Chim‐CD18‐WPRE to induce CD18 expression in mature granulocytes
No	Lung stem cell	N/A	Autologous	Idiopathic pulmonary fibrosis	N/A (Regend Therapeutics)	Injected via FOB	NCT02745184 (Phase 1/2)	None
No	EPC	N/A	Autologous	End‐stage liver disease	N/A (National University Hospital, Singapore)	Injected via PCD	NCT03109236 (Phase 3)	None
No	MSC	N/A	Allogeneic	Metabolic disease, endothelial dysfunction	N/A (Longeveron LLC)	IV	NCT02587572 (Phase 2)	None

Abbreviations: Cell subtypes: EPC, endothelial progenitor cell; MAPC, multipotent adult progenitor cell; NSC, neural stem cell. Indications: AIDS, acquired immunodeficiency syndrome; AML, acute myeloid leukemia; ADA, adenosine deaminase; ARDS, acute respiratory distress syndrome; aGvHD, acute graft versus host disease; CNS, central nervous system; COVID‐19, coronavirus disease 2019; COPD, chronic obstructive pulmonary disease; DOCK8 deficiency, dedicator of cytokinesis 8 deficiency; SCID, severe combined immunodeficiency. Routes: IA, intra‐articular; IV, intravenous; ICe, intracerebral; ITh, intrathecal; IP, intraperitoneal; IAe, intra‐arterial; FOB, fiberoptic bronchoscopy; PCD, percutaneous catheter. Note: Rev, regulator of expression of virion proteins; Tat, transactivator of expression.

#### 4.2.1 HSCs

HSCs comprise 44% of the current stem cell trials (Figure 4(a) **)**. These trials apply HSCT, which has been widely utilized in the clinic since it was first reported in 1957.^66^ The basic process for HSCT includes collection of mobilized stem cells from peripheral blood or cord blood, ex vivo purification and engineering of the cells, and finally infusion.^67^ Prior to transplantation, patients undergo a regimen of chemotherapeutics, broadly classified into myeloablative and reduced intensity conditioning (RIC), to deplete native lymphocytes in the bone marrow. This conditioning step is required for the successful engraftment of the infused cells.^68^ The choice of regimen depends on parameters such as disease severity, risk, age group, and other factors. RIC leads to reduced neutropenic periods, speedier engraftment, and improved recovery of the immune system compared to myeloablative conditioning. As a result, RIC has widened the potential applications of HSCT to a greater patient population, especially older patients.^69^ However, post‐transplant relapse and GvHD remain challenges, and these challenges are further discussed in Section 5.

HSCs are of great importance in the generation of a variety of blood cells and therefore have substantial implications in the genesis and treatment of hematological conditions.^70^ Accordingly, the majority of active HSC clinical trials (69%) are applied for various hematological malignancies and nonmalignant blood disorders associated with RBCs (BD w/RBCs) (Figure 4(a)), with remaining indications including solid cancers, nonmalignant blood disorders associated with white blood cells (BD w/WBCs), and immunodeficiency, autoimmune, and transplant‐related disorders. HSC source also plays a key role in the therapeutic outcome, and 61% of HSC trials use allogeneic cells (Figure 4(a)). This can be attributed to the fact that the most common goal of HSCT is to replace the patients' malignant immune cells with healthy donor HSCs, which are usually derived from a complete or partial HLA‐matched donor or “off‐the‐shelf” cord blood. On the contrary, GM HSC therapies apply autologous cells more frequently because disease‐causing mutations of patients' own cells are corrected by genetic engineering; autologous cells are associated with a lower risk of transplant rejection and thus are better tolerated by patients. In the following analysis, we present HSC trials according to disease indication and discuss the unique features of HSCs in each category.

The predominant indication of HSC trials is liquid cancers (51% of HSC trials). The majority of these trials (71%) use allogeneic cells that can differentiate into healthy blood cells and directly reconstitute the bone marrow (Figure 4(a) (i)). Notably, 12% of these trials are in Phase 3 or 4 (Figure 4(a) (ii)). This landscape of the Phase of HSC trials may reflect the application of well‐established HSCT methodology in nearly all trials, where only variations in pre‐ or post‐transplant therapeutic regimens are examined. On the other hand, GM HSCs are not yet FDA approved and are being investigated in the clinic for safety and efficacy. One example is a pair of trials using GM HSCs for the treatment of acquired immunodeficiency syndrome (AIDS)‐related liquid cancers (NCT02337985 and NCT01961063). Specifically, these HSCs were transduced with lentiviral vector rHIV7‐shI‐TAR‐CCR5RZ, with the mechanism of action including (i) blocking the transcription of HIV genes, (ii) binding to the protein that is essential for HIV replication, and (iii) catalyzing the CCR5 receptor that is required for viral entry into the host cells.^71^ This autologous therapy has the potential to reconstitute the bone marrow with autologous, non‐neoplastic cells.

The next focus of HSC trials is nonmalignant blood disorders associated with RBCs (BD w/RBCs) (18% of HSC trials) for diverse clinical indications including sickle cell disease, anemia, and thalassemia, among others. While NGM HSC trials mainly employ allogeneic cells (64%), GM HSC therapies exclusively employ autologous cells (36%) (Figure 4(a)(i) and (iii)). These autologous GM HSCs can be modified ex vivo to express correct sequences of target genes and thus generate cells with functional proteins. For example, the GM HSC product Zynteglo™ consists of autologous cells endowed with functional β‐globin by lentiviral transduction. It was approved for the treatment of transfusion‐dependent thalassemia by the EMA in 2019 and is currently being investigated for related indications including sickle cell disease (NCT04293185) and β‐thalassemia (NCT02906202). Another active area of HSC therapy is solid tumors (9% of HSC trials). Interestingly, 58% of these clinical trials utilize autologous cells (Figure 4(a)(i)), unlike the trials for blood cancers. This is not surprising because, in the case of solid tumors, the goal of HSC therapy is to bolster the existing immune system. Notably, all of the Phase 3 clinical trials for solid tumors (26%) are for the treatment of either brain metastases or neuroectodermal cancers (Figure 4(a)(ii)). The potential of HSCs to home to brain tumors has been shown in preclinical studies, although the mechanism remains unexplained.^72^

HSC‐based therapies are also clinically investigated for nonmalignant blood disorders associated with WBCs (BD w/WBCs) and immunodeficiency disorders, such as Wiskott‐Aldrich syndrome (WAS), severe combined immunodeficiency (SCID), and leukocyte adhesion disorder (LAD). 69% of these trials utilize GM HSCs, as the intention is to correct mutations responsible for disease (Figure 4(a)(iii)). While most of the trials (95%) are still in Phase 1 or 2 (Figure 4(a)(ii)), there is one trial in Phase 3, where elivaldogene autotemcel, a therapy that uses GM HSCs endowed with functional human adrenoleukodystrophy protein, is used to treat cerebral adrenoleukodystrophy (NCT03852498). HSCs are also being explored for the treatment of autoimmune disorders in a small subset of trials (4%) (Figure 4(a)). The specific indications are diverse and include multiple sclerosis (MS), systemic scleroderma, and Crohn's disease. While the number of trials in this area is low, the relative proportion of late stage trials is high; based on our analysis, over one‐third of the trials are in at least Phase 3 and one trial is in Phase 4. The majority of these late‐stage trials study the efficacy of autologous HSCT compared to the current clinical regimens, such as alemtuzumab for MS.

#### 4.2.2 MSCs

MSC therapies make up 46% of total current stem cell clinical trials (Figure 4(b)). MSCs are derived from multiple tissues, including bone marrow, adipose tissue, the umbilical cord, Wharton's jelly, and the placenta. Interestingly, the majority (65%) of the investigated MSC therapies are allogeneic, reflecting the trend of next‐generation “off‐the‐shelf” manufacturing (Figure 4(b)).^73^ While most of the investigated trials are in Phase 1 or 2, 6% of MSC trials have reached Phase 3 or 4. While GM cells are widely utilized in certain cell therapies (T cells in particular), the majority (> 98%) of the analyzed MSC trials use NGM MSCs. The widespread use of NGM cells may arise from their inherently multipotent nature, which enables a number of possible biological functions without genetic modification. Notably, four of the analyzed trials use GM MSCs, reflecting recent efforts to improve MSCs' innate functions or provide them with new functions such as secretion of therapeutic proteins. Current MSC trials cover a wide variety of indications, including degenerative diseases, autoimmune diseases, and cancer (Figure 4(b)). However, the majority of MSC trials can be broadly classified into two major categories based on their key mechanism: *tissue repair/regeneration* for degenerative diseases and trauma, and *immunomodulation* for autoimmune diseases, nonautoimmune inflammatory diseases, infectious diseases, and transplant‐related diseases. Trials that do not fall into these categories cover other indications such as cancer, cardiovascular diseases, and blood disorders.

The potential of MSCs to differentiate into cells across endodermal, mesodermal, and ectodermal lineages has been widely investigated in preclinical and clinical settings.^45^, ^47^, ^74^ Thus, a large portion of the current trials are focused on tissue repair or regeneration applications (32%), with the most prominent being degenerative diseases (24%) **(**Figure 4(b)). Based on our analysis, 30 trials use MSCs to repair cartilage for the treatment of osteoarthritis, which was one of the earliest indications for MSC therapies. As a representative example, JOINTSTEM, an autologous adipose‐derived MSC therapy, demonstrated suitable safety and preliminary efficacy profiles and is currently in two Phase 3 trials for osteoarthritis (NCT03990805, NCT04427930). Neurodegenerative disease is another particularly active area, with seven MSC trials underway for Alzheimer's disease, two for Parkinson's disease, and three for Huntington's disease. Based on our analysis, all but one of these neurodegenerative disease trials are still in early stages (Phase 1 or 2), with one exception reaching Phase 2/3. This Phase 2/3 trial involves the autologous MSC therapy, Cellavita‐HD, which has previously demonstrated promising safety and preliminary efficacy profiles for Huntington's disease (NCT04219241). Other degenerative disease indications of MSC therapy include disk degeneration, aging frailty, and several others. Trauma is another important indication for MSC tissue repair applications, with 23 active trials (8%) (Figure 4(b)) underway for traumatic brain injury, spinal cord injury, acute kidney injury, ischemia reperfusion injury, and others. While the therapeutic mechanism of MSCs in these degenerative diseases seems to be related to their multipotent differentiation capabilities, their immunomodulatory potential may also play an important role.^75^

Immunomodulatory capabilities of MSCs can also be leveraged as the primary mechanism for disease management.^45^, ^76^, ^77^, ^78^ About 49% of the analyzed MSC trials are focused on this application for the treatment of immune‐related diseases. Autoimmune diseases, as the second largest disease category of all MSC therapies (18%) (Figure 4(b)), encompass rheumatoid arthritis, type 1 diabetes, MS, systemic lupus erythematosus, and others. Although all of these trials are in the early stages (Phase 1 or 2), the large number of trials is indicative of the overall promise of MSCs for autoimmune disease applications. Non‐autoimmune inflammatory disease is another important area for MSC immunomodulation clinical studies, with 14% of all MSC trials (Figure 4(b)) underway for the treatment of bronchopulmonary dysplasia, liver cirrhosis, acute and chronic pancreatitis, chronic obstructive pulmonary disease (COPD), and glomerulonephritis. About 13% of active MSC trials are for infectious diseases (Figure 4(b)). With the ongoing COVID‐19 pandemic, MSC‐based therapies have already entered the clinic for the treatment of SARS‐CoV‐2 infections, with two trials having already entered Phase 2/3. As a representative example, Remestemcel‐L is an allogeneic MSC product that was initially investigated for pediatric aGvHD and is now being repurposed for the treatment of COVID‐19 ARDS in a Phase 3 study (NCT04371393). MSC therapy for transplant‐related diseases also leverages the immunomodulatory capacity of MSCs, with 4% of all MSC trials underway to treat aGvHD and organ transplant rejection, particularly of the liver, kidney, and lungs (Figure 4(b)).

Most of the clinical efforts so far have been mainly focused on employing the multipotent capability of NGM MSCs for diverse indications. However, in recent years, extensive preclinical studies have looked into GM MSCs, especially for the indication of cancer.^47^ Indeed, MSCs have been engineered to produce anticancer therapeutic proteins, such as TNF‐related apoptosis‐inducing ligand (TRAIL) and thrombospondin‐1, in preclinical studies.^47^, ^79^, ^80^, ^81^, ^82^ These new advances are reflected in the clinical trial landscape, where allogeneic MSCs engineered to express TRAIL (MSCTRAIL) are currently being investigated as a therapeutic for inoperable lung adenocarcinomas (NCT03298763).

#### 4.2.3 Other stem cells

Other types of stem cells account for only 10% of all current clinical stem cell trials, far fewer trials than either HSCs or MSCs (Figure 4(c–e)). These stem cells include NSCs (Figure 4(c)), bone marrow‐derived stem cells (Figure 4(d)), cardiac stem cells, endothelial progenitor cells, LSCs, and multipotent adult progenitor cells, among others (Figure 4(e)). The relative rarity of these stem cell types in the clinic compared to HSCs and MSCs may be due to a variety of factors such as (i) the biological functions of these stem cells are not as diverse as those of HSCs and MSCs^83^ and (ii) availability/manufacturing of these stem cells are more challenging.^84^ Of note, the investigated applications for these subtypes are relatively narrow. For example, specific indications of NSCs include astrocytoma, glioma, Parkinson's disease, retinitis pigmentosa, and spinal cord injury. Notable applications of other subtypes include lung stem cells for idiopathic lung fibrosis and interstitial lung diseases, retinal progenitor cells for retinitis pigmentosa, and multipotent adult progenitor cells for immunomodulatory applications.

### 4.3 Dendritic cells

**FIGURE 5:**
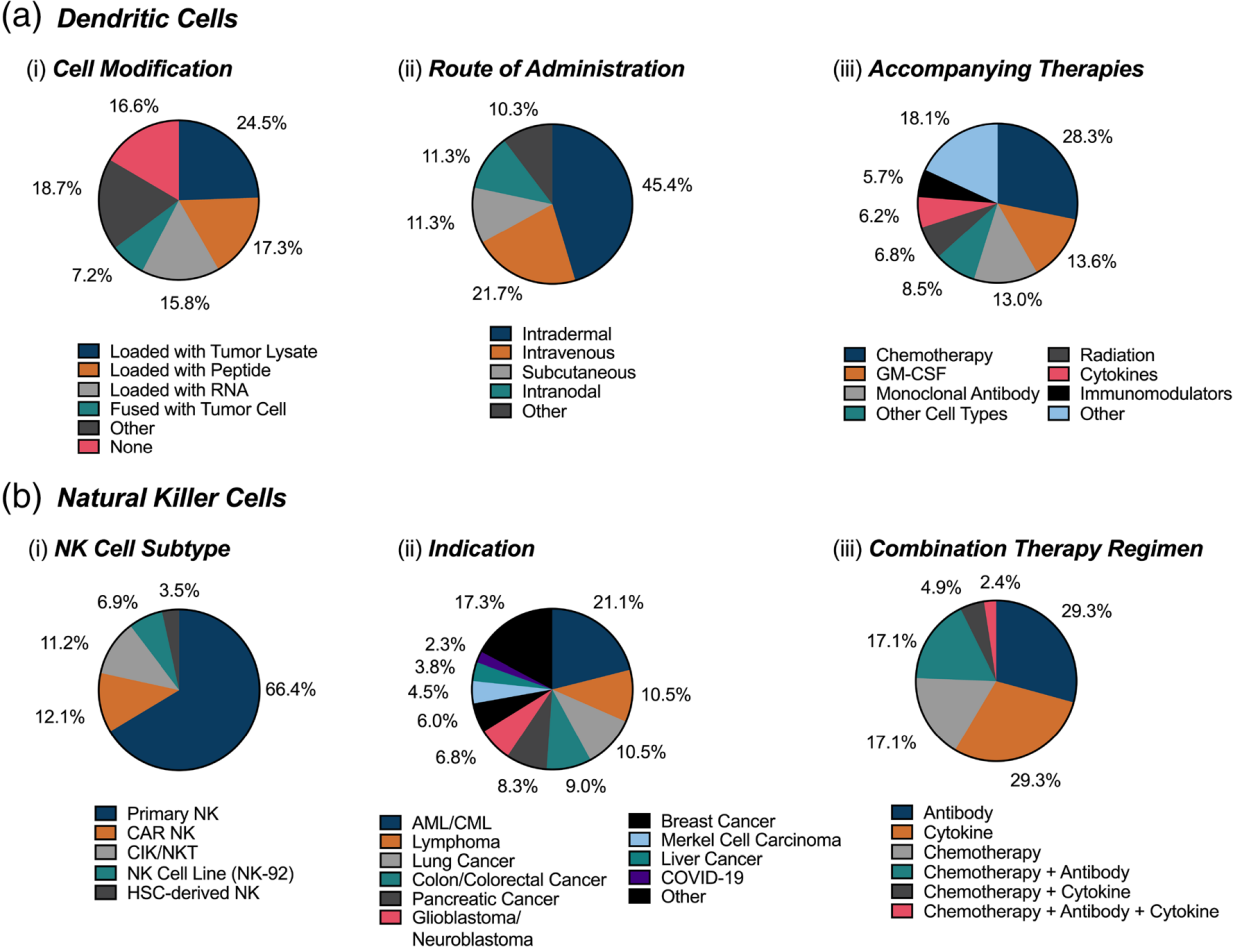
Current landscape of dendritic cell and natural killer cell clinical trials. (a) About 136 clinical trials using DCs were further classified according to DC modifications, route of administration, and accompanying therapies; (b) 116 trials using NK‐cell therapies were further classified according to indication, NK cell subtype, and combination therapy regimen. mRNA: messenger RNA; GM‐CSF: granulocyte‐macrophage colony‐stimulating factor; AML/CML: acute/chronic myeloid leukemia; CAR: chimeric‐antigen receptor; CIK: cytokine‐induced killer cells; NKT: natural killer T cells

DC vaccines are an active area of clinical study, with 93% of trials investigating their use for cancer immunotherapy (Figure 2). A representative selection of DC trials is shown in Table 4. DC clinical trials have applications for a range of both solid and liquid tumors (93%) and the remainder for autoimmune disorders, infectious diseases, and transplant‐related disorders. Based on our analysis, all Phase 3 trials and most Phase 1 and 2 trials are for cancer indications, although 6% of the current Phase 1 and 2 trials investigate DC vaccines for the treatment of multiple sclerosis, type I diabetes, rheumatoid arthritis, HIV, COVID‐19, and transplant‐related diseases. Like the approved products (three globally, with one in the United States), the majority of DC trials (89%) use autologous cells **(**Figure 2 **)**. However, allogeneic cells have demonstrated potential for indications such as leukemia (NCT03679650) and living donor liver transplant (NCT04208919). The trial space for DCs is generally diverse, owing to the investigation of many different ex vivo cell pretreatments. Ex vivo pretreatment of DCs primarily involves antigen priming, which is necessary to expose the cells to tumor antigens so that they can present antigen‐specific peptides on MHC class II to the patient's T cells in the lymph nodes. The most common method for exposing the cells to tumor antigens is ex vivo incubation and loading (also frequently termed “pulsing”) with specific antigens, tumor lysate, or RNA. Another novel method involves direct fusion of the DCs with patient‐specific tumor cells obtained via a biopsy. In six clinical trials involving RNA delivery, electroporation is used to promote ex vivo loading in DCs. These cell modifications are summarized in Figure 5(a) (i).

**TABLE 4:** Examples of current clinical trials for dendritic cell therapies, grouped by indication

GM	Priming materials	Source	Indication	Name (Sponsor)	Route	Accompanying therapies	Trial number	Other trials and/or notes
*Cancer (n = 127)*								
No	Tumor‐specific antigens	Autologous	Glioblastoma	N/A (NeuroVita Clinic)	SC	(co) HSCs, cytotoxic lymphocytes, GM‐CSF	NCT01759810 (Phase 2/3)	None
Yes	Tumor RNA	Autologous	Uveal melanoma	N/A (University Hospital Erlangen)	IV	N/A	NCT01983748 (Phase 3)	None
Yes	pp65‐LAMP mRNA	Autologous	Glioblastoma	N/A (Duke University Medical Center)	ID	(pre) Tetanus, basilixumab, (post) Temozolomide	NCT02366728 (Phase 2)	None
No	Tumor cell lysate	Autologous	Metastatic colorectal cancer	APDC (Second Military Medical University)	IV	(co) Chemotherapy (oxaliplatin, 5‐fluorouracil, leucovorin)	NCT02503150 (Phase 3)	None
Yes	TT‐RNA	Unknown	Glioma	TTRNA‐DC (University of Florida)	ID	(pre) Chemotherapy (temozolomide), (co) GM‐CSF, tetanus, autologous HSCs	NCT03334305 (Phase 1)	NCT03396575 (Phase 1)
No	Tumor cell lysate	Autologous	Mesothelioma	MesoPher (Amphera B.V.)	Unk	(co) Best supportive care	NCT03610360 (Phase 2/3)	None
No	PA2024	Autologous	Prostate adenocarcinoma	Sipuleucel‐T (Dendreon)	IV	(co) GM‐CSF	NCT03686683 (Phase 3)	None
No	Synthetic peptides	Cell line	Non‐small cell lung cancer	PDC*lung01 (PDC*line Pharma SAS)	IV	(pre) Radiation, (post) Pemetrexed	NCT03970746 (Phase 1/2)	None
No	Tumor‐specific antigens	Autologous	Glioblastoma	ADCTA (Safe Save Medical Cell Sciences & Technology Co., Ltd.)	SC	(co) Bevacizumab	NCT04277221 (Phase 3)	None
No	AML cells	Allogeneic	AML	N/A (Dana Farber Cancer Institute)	SC	(co) GM‐CSF, (post) Decitabine	NCT03679650 (Phase 1)	None
No	Loaded with tumor homogenate	Autologous	Head and neck tumors, neuroendocrine tumors, soft tissue sarcoma	N/A (Istituto Scientifico Romagnolo per lo Studio e la cura dei Tumori)	ID	(post) IL‐2	NCT04166006 (Phase 2)	None
No	Fused with myeloma cells	Autologous	Multiple myeloma	N/A (Beth Israel Deaconess Medical Center)	Unk	(co) Anti‐PD‐1	NCT01067287 (Phase 2)	None
No	None	Autologous	Follicular lymphoma	N/A (Oslo University Hospital)	IN	(pre) Radiation, anti‐PD‐L1 immunotherapy, (co) Rituximab, GM‐CSF, pembrolizumab, (post) Pembrolizumab	NCT02677155 (Phase 2)	None
Transplant‐related diseases (*n* = 3)								
No	N/A	Allogeneic	Liver transplantation	DCreg (University of Pittsburgh)	IV	Standard‐of‐care immunosupression, weaned after 1 week	NCT04208919 (Phase 1/2)	Usage of DCreg
*Infectious Diseases (n = 3)*								
No	SARS‐CoV‐2 antigen	Autologous	COVID‐19	AV‐COVID‐19 (Aivita Biomedical, Inc.)	SC	(co) GM‐CSF	NCT04386252 (Phase 1/2)	None
Autoimmune diseases (*n* = 3)								
No	Myelin‐derived peptide	Autologous	Multiple sclerosis	toIDC (Antwerp University Hospital)	ID	N/A	NCT02618902 (Phase 1)	None

Abbreviations: Routes: ID, intradermal; IN, intranodal; IV, intravenous; SC, subcutaneous; Unk, unknown; . Priming materials: AML, acute myeloid leukemia; pp65‐LAMP mRNA, phosphoprotein 65 lysosomal associated membrane protein messenger RNA; TT‐RNA, total tumor RNA; PAP, GM‐CSF fusion protein, PA2024; SARS‐CoV‐2, severe acute respiratory syndrome coronavirus 2. Indications: COVID‐19, coronavirus disease 2019. Accompanying therapies: GM‐CSF, granulocyte‐macrophage colony‐stimulating factor; PD‐1, programmed cell death protein 1; PD‐L1, programmed death‐ligand 1.

Other distinguishing aspects of the DC vaccine trial space include the routes of administration, addition of immune stimulatory molecules, and other combination drug regimens. The most common route of administration for DC vaccines is intradermal injection (45%), while intravenous (22%), subcutaneous (11%), and intranodal (11%) injections are also used (Figure 5(a) (ii)). Co‐administered stimulatory molecules including growth factors, cytokines, and monoclonal antibodies can promote the expansion and trafficking of DCs to the lymph node (e.g., GM‐CSF) (NCT03334305), activate T and NK cells (e.g., IL‐2) (NCT04166006), or sensitize cancer cells to cytotoxic T cells (e.g., anti‐PD‐1, anti‐PD‐L1) (NCT01067287, NCT02677155) (Figure 5(a) (iii)). Other common accompanying therapies include chemotherapeutics (NCT02366728, NCT02503150), other cell types (e.g., stem cells (NCT03334305), T cells (NCT01759810), immunomodulators (NCT04208919), and radiation (NCT03970746).

### 4.4 NK cells

NK cells have drawn increased attention over the past few years due to their natural cytotoxic functions,^85^ non‐MHC restricted activity, and “off‐the‐shelf” use capability.^86^, ^87^ These features allow them to offer a potent alternative to T‐cell therapy. Currently, NK cell trials occupy 7% of total cell therapy trials (Figure 2), with a representative selection of these trials listed in Table 5.

**TABLE 5:** Examples of current clinical trials for natural killer cell therapies, grouped by indication

GM	Sub‐type	Target	Source	Indication	Name (Sponsor)	Route	Accompanying therapies	Trial number	Other trials and/or notes
Liquid cancer (*n* = 42)									
Yes	CAR	CD19	Allogeneic	Lymphoma	N/A (M.D. Anderson Cancer Center)	IV	None	NCT03056339 (Phase 1/2)	Suicide gene
No	N/A	N/A	Allogeneic	Lymphoma	N/A (M.D. Anderson Cancer Center)	IV	None	NCT04074746 (Phase 1)	AFM13 bispecific antibody delivered as NK cell payload
No	N/A	N/A	Allogeneic	Lymphoma	MG4101 (Green Cross LabCell Corporation)	IV	(co) IL‐2, rituximab	NCT03778619 (Phase 1/2)	None
No	N/A	N/A	Allogeneic	AML	N/A (M.D. Anderson Cancer Center)	IV	None	NCT01787474 (Phase 1/2)	None
No	N/A	N/A	Allogeneic	AML	UCB‐NK (Radboud University)	IV	(co) IL‐2	NCT04347616 (Phase 1/2)	None
No	N/A	N/A	Allogeneic	AML, MDS	N/A (Washington University School of Medicine)	IV	(co) IL‐2	NCT01898793 (Phase 1/2)	None
No	NK‐92	N/A	Cell line	Leukemia, lymphoma, MDS	N/A (M.D. Anderson Cancer Center)	IV	None	NCT02727803 (Phase 2)	None
No	N/A	N/A	Allogeneic	AML	N/A (St. Jude Children's Research Hospital)	IV	None	NCT00703820 (Phase 3)	None
Solid cancer (*n* = 68)									
No	CIK/NKT	N/A	Autologous	Glioma	N/A (The First People's Hospital of Changzhou)	IV	(co) Temozolomide	NCT02496988 (Phase 4)	None
Yes	CAR NK‐92	HER2	Cell line	Glioblastoma	NK‐92/5.28.z (Johann Wolfgang Goethe University Hospital)	ICr	None	NCT03383978 (Phase 1)	None
No	N/A	N/A	Allogeneic	Glioblastoma	CYNK‐001 (Celularity, Inc.)	IV, IT	None	NCT04489420 (Phase 1)	None
No	N/A	N/A	Autologous	Hepatocellular carcinoma	IKC (Ivy Life Sciences, Co., Ltd)	IV	None	NCT03592706 (Phase 2/3)	None
No	CIK/NKT	N/A	Autologous	Cholangiocarcinoma	N/A (The First People's Hospital of Changzhou)	IV	None	NCT02482454 (Phase 2/3)	None
No	NK‐92	N/A	Cell line	Merkel cell carcinoma	aNK (NantKwest, Inc.)	IV	(co) ALT‐803	NCT02465957 (Phase 2)	None
No	N/A	N/A	Autologous	HER2/EGFR+ cancer	SNK01 (NKMax America, Inc.)	IV	(co) Trastuzumab, cetuximab	NCT04464967 (Phase 1/2)	None
No	N/A	N/A	Autologous	Solid tumors	N/A (Xuanwu Hospital, Beijing)	IV	None	NCT03634501 (Phase 1/2)	None
Infectious diseases (*n* = 5)									
Yes	CAR	SARS‐CoV‐2 S protein, NKG2DL	Allogeneic	COVID‐19	N/A (Chongqing Public Health Medical Center)	IV	None	NCT04324996 (Phase 1/2)	Secrete IL‐15 superagonist and anti‐GM‐CSF scFv
No	N/A	N/A	Allogeneic	COVID‐19	CYNK‐001 (Celularity, Inc.)	IV	None	NCT04365101 (Phase 1/2)	None
No	N/A	N/A	Allogeneic	COVID‐19	N/A (Xinxiang Medical University)	IV	None	NCT04280224 (Phase 1/2)	None

Abbreviations: Cell Subtypes: CAR, chimeric antigen receptor; CIK, cytokine‐induced killer; NK‐92, immortal NK cell line; NKT, natural killer T. Targets: CD, cluster of differentiation; SARS‐CoV‐2, severe acute respiratory syndrome coronavirus 2. Indications: AML, acute myeloid leukemia; COVID‐19, coronavirus disease 2019; EGFR, endothelial growth factor receptor; HER2, human epidermal growth factor receptor 2; MDS, myelodysplastic syndrome. Routes: IV, intravenous; ICr, intracranial; IT, intratumoral. Payloads: iCasp9, inducible caspase 9; CD19t, truncated CD19. Accompanying Therapies: IL‐2, interleukin 2. IL‐15, Note: interleukin 15; scFv, single‐chain variable fragment.

NK cell trials utilize NK cells of various origins, including allogeneic sources (53%), autologous sources (27%), NK cell lines (15%), and stem cell‐derived (allogeneic) sources (5%) (Figure 2). The frequent use of allogeneic NK cells may be due to the therapeutic effects associated with the killer‐immunoglobulin receptor (KIR)‐HLA mismatch.^88^ Specifically, the identification of self‐MHCs by KIRs provides inhibitory signals to NK cells to halt cytotoxic functions; in the case of adoptive transfer of allogeneic cells, these signals are absent. In addition, when allogeneic NK cells simultaneously encounter foreign MHCs and disease‐associated antigens on target cells, activation signals are triggered. This combination of missing inhibitory signals and multiple activating signals elicits the cytotoxic function of allogeneic NK cells against diseased cells. Although peripheral blood NK cells are still the major source in NK‐cell therapy (80%, combined autologous and allogeneic) (Figure 2), there are translational challenges due to limited availability (i.e., NK cells only account for 5%–10% of peripheral white blood cells) and difficulty of expansion ex vivo. Induced pluripotent stem cell (iPSC)/HSC‐derived cell products and cell lines have been introduced in the clinic to tackle these challenges by providing a sufficient cell source and eliminating the barrier of ex vivo expansion.

Several NK‐cell subtypes are applied in NK‐cell trials, encompassing primary NK cells, CAR‐NK cells, CIKs, NK cell lines (NK‐92), and iPSC/HSC‐derived NKs (Figure 5(b) (i)). Most NK cell trials (66%) use unmodified primary NK cells and leverage their intrinsic cytotoxic function (Figure 5(b) (i)). CAR‐NK therapies (12%) have gained attention over the past few years due to encouraging results following the recent approval of CAR‐T products (Figure 5(b) (i)). CAR‐NK cells are generally regarded as safer than CAR‐T cells due to their more limited persistence in circulation.^89^ CAR‐NK cells are also less likely to induce severe side effects because CAR‐NK cells usually produce IFN‐γ and GM‐CSF upon activation, while CAR‐T cells produce a set of cytokines (IL‐1a, IL‐2, IL‐6, TNF‐α, MCP‐1, IL‐8, IL‐10, IL‐15, and others) that have been shown to cause cytokine release syndrome and severe neurotoxicity.^89^ CAR‐NK cell therapies are used for (i) hematological malignancies expressing CD19 (NCT03056339), BCMA, and CD 22; (ii) solid tumors with targets including ROBO‐1, PSMA, and mesothelin; and (iii) COVID‐19 with ACE‐2 as a target (NCT04324996). Another major cell class is CIKs (11%) (Figure 5(b) (i)), which have an NK‐T hybrid cell phenotype and operate in a non‐MHC restricted fashion.^90^ CIKs are also referred to as type II NKT cells.^91^ An attractive feature of CIKs is that they can be induced and expanded ex vivo from PBMCs and cord blood mononuclear cells (CBMCs) via a combination of cytokines.^90^ Currently, one of the most advanced NK cell trials for malignant glioma (Phase 4) uses CIKs as the intervention (NCT02496988). The NK‐92 cell line (7%) is another promising candidate due to its ease of expansion and transfection in comparison to primary NK cells (Figure 5(b) (i)).^92^ It has been validated as safe in many Phase 1 trials^92^ and is being currently used in advanced trials (NCT02727803, NCT02465957). Finally, iPSC/HSC‐derived cells (4%) are being explored to overcome the limit of NK cell availability from PBMCs (Figure 5(b) (i)). For example, CYNK‐001, an HSC‐derived NK product, is currently being investigated in a Phase 1/2 clinical trial for the treatment of COVID‐19 (NCT04365101).

The main indication of NK‐cell therapy is cancer (95%), followed by infectious diseases (e.g., COVID‐19 and HIV) (4%) (Figure 2). A large proportion of NK‐cell trials are for hematologic malignancies, with 21% for acute/chronic myeloid leukemia (AML/CML) and 11% for lymphoma (Figure 5(b) (ii)). Notably, despite limited information regarding tumor infiltration by NK cells,^93^ there are more clinical trials investigating NK‐cell therapy for solid cancers than liquid cancers. These trials against solid tumors often use CAR and combination therapy approaches to improve targeting and overcome the immunosuppressive tumor microenvironment in cancers such as glioblastoma (NCT03383978).^94^, ^95^ Interestingly, based on our analysis, augmenting NK‐cell therapy with co‐administered or postadministered therapeutics is a strategy adopted by a considerable portion of the NK trials (35%). Antibodies (NCT04074746) and cytokines (NCT03050216) are the most used supporting therapeutics (29% for both), followed by chemotherapeutics (NCT02496988), and a combination of those (NCT03778619) (Figure 5(b) (iii)).

### 4.5 Mononuclear cells

Compared to the other cell types, there are fewer clinical trials for mononuclear cells (2% of the total trials) (Figure 2). As stated previously, we refer to mononuclear cells as belonging to one of the following cell populations: monocytes (11% of mononuclear cell trials), macrophages (0% of mononuclear cell trials), BMMCs (52% of mononuclear cell trials), or PBMCs (37% of mononuclear cell trials) (Table 6). The most common indications are cardiovascular disease (39%) and cancer (29%), with some applications for trauma (14%) (Figure 2 **)**. These trials are almost evenly distributed across Phase 1 (38%), Phase 2 (33%), and Phase 3 (30%) (Figure 2). Most (89%) of these trials use autologous cells, likely due to the highly regulated immune functions of these cell types (Figure 2).

**TABLE 6:** Examples of current clinical trials for mononuclear cell therapies, grouped by indication and cell type

Cell Type	GM	Cell Modifications	Source	Indication	Name (Sponsor)	Route	Accompanying therapies	Trial number	Other trials and/or notes
Cancer (*n* = 8)									
Monocytes	No	Stimulated with IFNs (Actimmune®, Sylatron™)	Autologous	Fallopian tube, ovarian, and peritoneal cancers	N/A (National Cancer Institute)	IP	N/A	NCT02948426 (Phase 1)	None
PBMC	No	Depletion of naive (CD45RA+) T cells	Allogeneic	GvHD, infection	N/A (Federal Research Institute of Pediatric Hematology, Oncology and Immunology)	IV	N/A	NCT02942173 (Phase 2/3)	None
PBMC	Yes	siRNA‐transfected	Autologous	Solid tumors	APN401 (Wake Forest University Health Sciences)	IV	N/A	NCT03087591 (Phase 1)	siRNA silencing of cbl‐b, an E3 ubiquitin ligase, to enhance activation of lymphocytes
PBMC	Yes	Express NY‐ESO‐1 TCR	Autologous	Malignant neoplasms	N/A (Jonsson Comprehensive Cancer Center)	IV	NY‐ESO‐1 peptide‐pulsed dendritic cells (DC vaccine), IL‐2	NCT01697527 (Phase 2)	None
PBMC	No	Depletion of TCR‐αβ T cells and B cells, enrichment of NK cells and TCRγδ T cells	Allogeneic	AML, ALL	N/A (Case Comprehensive Cancer Center)	IV	N/A	NCT03939585 (Phase 1)	None
Cardiovascular diseases (*n* = 11)									
BMMC	No	N/A	Autologous	Heart failure	t2c001 (Johann Wolfgang Goethe University Hospital)	ICI	N/A	NCT01693042 (Phase 2/3)	None
BMMC	No	N/A	Autologous	Heart failure (post‐myocardial infarction)	N/A (American Heart of Poland)	ICI	N/A	NCT02323620 (Phase 3)	None
BMMC	No	N/A	Autologous	Critical limb ischemia	REX‐001 (Rexgenero Limited)	IAe	N/A	NCT03111238 (Phase 3)	None
Trauma (*n* = 4)									
BMMC	No	N/A	Autologous	Traumatic brain injury	N/A (The University of Texas Health Science Center, Houston)	IV	N/A	NCT01851083 (Phase 1/2)	None
BMMC	No	N/A	Autologous	Spinal cord injury	N/A (Shanghai Changzheng Hospital)	ITh	N/A	NCT04528550 (Phase 2)	None
Infectious diseases (*n* = 1)									
PBMC	No	Stem cell educated (co‐cultured with adherent human multipotent cord blood stem cells in vitro)	Autologous	COVID‐19	N/A (Tianhe Stem Cell Biotechnologies Inc.)	IV	N/A	NCT04299152 (Phase 2)	Stem Cell Educator (SCE) co‐culture designed to induce tolerance in T cells responsible for inflammation
Other (*n* = 3)									
BMMC	No	N/A	Autologous	Type 2 diabetes	N/A (Van Hanh General Hospital)	IV	Adipose mesenchymal cells	NCT03943940 (Phase 1/2)	None

Abbreviations: Cell subtypes: BMMC, bone marrow mononuclear cells; PBMCs, peripheral blood mononuclear cells. Cell modifications: IFNs, interferons; NY‐ESO‐1, New York esophageal squamous cell carcinoma‐1; siRNA, small interfering RNA. Indications: AML, acute myeloid leukemia; ALL, acute lymphocytic leukemia; COVID‐19, coronavirus disease 2019; GvHD, graft versus host disease. Routes: IP, intraperitoneal; IV, intravenous; IT, intratumoral; ICI, intracoronary infusion; IAe, intra‐arterial; ITh, intrathecal. Accompanying therapies: IL‐2, interleukin 2; NY‐ESO‐1, New York esophageal squamous cell carcinoma‐1.

There are many preclinical studies that use monocytes and macrophages as therapies, therapeutic targets, or both, most often for the treatment of cancer, autoimmune diseases, and other inflammatory diseases.^96^, ^97^, ^98^, ^99^ However, translation to the clinic has been limited. For monocytes, two trials are indicated for central nervous system (CNS) disorders, while one trial is indicated for cancer. Notably, there are a number of studies where monocytes are separated via leukapheresis and subsequently differentiated into DCs for re‐injection. Due to their highly plastic nature, monocytes are well‐suited for ex vivo conditioning, which is used to induce phenotypic changes before re‐injection (NCT02948426). While many trials use macrophage populations as a target for imaging agents or as an indicator of clinical outcomes, these cells are not currently being infused as an intervention.

BMMCs and PBMCs were also included in the clinical trial analysis. These groups are clinically useful because they invoke pleiotropic mechanisms, owing to the variety of distinct cell populations that are included. This allows, for example, the secretion of various remodeling factors to facilitate wound healing and regeneration.^100^ Similar to monocytes/macrophages, ex vivo conditioning allows for the induction of phenotypic changes (NCT02948426). It is also possible to enrich or deplete these mononuclear cells of certain cell populations, such as naïve T cells (NCT02942173) or B cells (NCT03939585). Other modifications include transfection with small interfering RNA (siRNA) (NCT03087591) and induction of specific receptors before re‐infusion (NCT01697527). Combination therapies with stem cells are also being investigated (NCT03943940). Though the trial space for mononuclear cells is in its early stages, the multifunctional properties of these cells make them an attractive avenue for a diverse array of future therapies.

### 4.6 Red blood cells and platelets

Clinical trials are also being conducted on the intravenous infusion of donor blood products, such as packed RBCs, whole blood, and platelets. Here, we focus our scope on the trials that use a single cell type, either RBCs (2% of the total trials) or platelets (0.4% of the total trials) (Figure 2). A representative selection of these trials is given in Table 7. Of all the clinical trials involving RBCs, the most common indications are blood disorders such as anemia (26%) and sickle cell disease (18%). In the majority of these trials, as well as those focused on treating hemorrhage, organ injury, and trauma, the unmodified RBCs themselves are the therapeutic of interest. While packed RBCs are already indicated for these conditions, current clinical trials investigate new infusion protocols (e.g., dosing frequency and amount), cell storage conditions, additional ex vivo cell processing steps for pathogen removal, and D‐antibody matching.

**TABLE 7:** Examples of current clinical trials for red blood cell (RBCs) and platelet therapies, grouped by indication and cell type

Cell type	GM	Source	Indication	Name (Sponsor)	Route	Accompanying therapies	Trial number	Other trials and/or notes
Blood disorders (*n* = 17)								
RBC	No	Allogeneic	Anemia	N/A (St. Justine Hospital Center)	IV	None	NCT03871244 (Phase 3)	None
Metabolic disorders (*n* = 3)								
RBC	No	Allogeneic	Glucose transporter type 1 deficiency syndrome	N/A (University of Texas Southwestern Medical Center)	IV	Glucose transporter 1 (GLUT‐1), expressed endogenously on donor RBCs	NCT04137692 (Early Phase 1)	None
RBC	No	Autologous	Mitochondrial neurogastrointestinal encephalomyopathy	EE‐TP (St. George's, University of London)	IV	*E. coli* thymidine phosphorylase, encapsulated in RBC	NCT03866954 (Phase 2)	None
RBC	Yes	Allogeneic	Phenylketonuria	RTX‐134 (Rubius Therapeutics)	IV	AvPAL (*Anabaena variabilis* phenylalanine ammonia lyase), expressed intracellularly	NCT04110496 (Phase 1)	None
Cancer (*n* = 4)								
RBC	No	Allogeneic	Triple negative breast cancer	Eryaspase (ERYtech Pharma)	IV	l‐asparaginase, encapsulated in RBC	NCT03674242 (Phase 2/3)	NCT01518517 (Phase 2/3)
RBC	No	Unknown	Non‐small cell lung cancer	N/A (State Key Laboratory of Respiratory Disease)	IV	*P. vivax* parasite, via infection of RBC	NCT02786589 (Phase 1/2)	NCT04165590 (Phase 1/2), NCT03474822 (Phase 1/2), NCT03375983 (Phase 1/2)
Infectious diseases (*n* = 1)								
RBC	No	Allogeneic	Malaria	N/A (University of Oxford)	IV	*P. vivax* parasite, via infection of RBC	NCT03797989 (Early Phase 1)	None
Other (*n* = 4)								
RBC	No	Autologous	Ataxia telangiectasia	EryDex (Erydel)	IV	Dexamethasone sodium phosphate, encapsulated in RBC	NCT03563053 (Phase 3)	NCT02770807 (Phase 3)
Trauma (*n* = 4)								
Platelet	No	Allogeneic	Surgical blood loss, hemorrhage	N/A (Australian and New Zealand Intensive Care Research Centre)	IV	None	NCT03991481 (Phase 3)	Efficacy and safety of cryopreserved vs. liquid platelets
Blood disorders (*n* = 3)								
Platelet	No	Allogeneic	Thrombocytopenia associated with hematological malignancies	N/A (Emory University)	IV	None	NCT02074436 (Phase 2)	Comparison to epsilon aminocaproic acid (EACA)

Abbreviation: Routes: IV, intravenous.

RBCs are also indicated in other Phase 1 or Phase 2 trials with indications of cancer, lung cancer, breast cancer, and malaria infection, the latter of which involves RBCs infected ex vivo with the parasite *Plasmodium vivax* (*P. vivax*). These infected RBCs are administered to malaria‐naïve volunteers to evaluate the safety of controlled blood‐stage human *P. vivax* malaria infection and are expected to serve as a malaria vaccine in the future (NCT03797989). Notably, in seven trials, RBCs (two autologous and five allogeneic) are being used as drug carriers to carry and deliver membrane‐bound (NCT04137692) or encapsulated cargoes (NCT03563053) to a site of interest. In all RBC trials, the RBCs and associated cargoes are administered intravenously. Platelet transfusions are investigated clinically for thrombocytopenia and for the induction of blood clotting following trauma. In all these trials, the platelets are administered intravenously. As with RBCs, new dosing regimens for platelet transfusions are also investigated. One distinct application of platelets in clinical trials is their co‐administration alongside a thrombolytic drug in platelet‐deficient patients (NCT02074436).

### 4.7 Microbes

Forty‐eight microbe‐based clinical trials were identified (3% of the total trials) (Figure 2) and separated into four main categories based upon consortia‐based/single strain‐based therapy status and indication (Table 8).^4^, ^33^, ^101^ Undefined, consortia‐based therapies consist of microbes collected from healthy patient stool banks.^101^ The resulting microbe therapeutic ranges from unpurified material stripped only of harmful components (e.g., viruses, pathogens) to material stripped of several species in order to isolate specific microbial phyla with some, or all, individual species remaining unidentified. These undefined mixtures are most similar to those used in FMT, however, the therapeutic is typically administered via oral capsules.^102^, ^103^ Since FMTs fall under the category of a clinical procedure^101^ and are not being developed as drug products by independent pharmaceutical companies, FMT clinical trials were not included, as we determined them to be out of the scope of this review (a search on clinicaltrials.gov revealed over 900 hits for the term “fecal microbiota transplant”). Defined, consortia‐based therapies are similar in that multiple strains of bacteria are orally delivered; however, the exact composition of the individual microbial species is known and is rationally selected. Many of the current consortia‐based clinical trials are for *Clostridium difficile* infection and various gastrointestinal disorders. Consortia‐based therapies make up 44% of the current microbe clinical trials with 25% being defined consortia and 19% being undefined consortia **(**Figure 2).

**TABLE 8:** Examples of current clinical trials for microbe therapies, grouped by indication

GM	Sub‐type	Cell modifications	Source	Indication	Name (Sponsor)	Route	Accompanying therapies	Trial number	Other trials and/or notes
*Consortia for Gut Microbiota (n = 20)*									
No	Donor derived	N/A	UMC	*Clostridium difficile* infection	CP101 (Finch Therapeutics)	Oral	None	NCT03110133 (Phase 2)	NCT03497806 (Phase 2), NCT04182633 (Phase 2)
No	Donor‐derived bacterial spores	N/A	UMC	Clostridium difficile infection	SER‐109 (Seres Therapeutics)	Oral	None	NCT03183128 (Phase 3)	NCT03183141 (Phase 3)
No	Donor‐derived bacterial spores	N/A	UMC	Ulcerative colitis	SER‐287 (Seres Therapeutics)	Oral	None	NCT03759041 (Phase 2)	NCT03817125 (Phase 1)
No	Donor derived	N/A	UMC	Clostridium difficile infection	RBX7455 (Mayo Clinic/Rebiotix Inc.)	Oral	None	NCT02981316 (Phase 1)	NCT04139993 (Phase 1), NCT04155099 (Phase 2)
No	11 donor‐derived bacterial strains	N/A	DMC	Advanced or metastatic cancer	VE800 (Vedanta Biosciences/Bristol‐Myers Squibb)	Oral	(co) Nivolumab	NCT04208958 (Phase 1/2)	NCT03936998 (Phase 1/2)
No	8 donor‐derived bacterial strains	N/A	DMC	Clostridium difficile infection	VE303 (Vedanta Biosciences)	Oral	None	NCT03788434 (Phase 2)	None
No	26 donor‐derived bacterial strains	N/A	DMC	Obesity/hypertriglyceridemia	MET 5 (NuBiyota)	Oral	None	NCT04507971 (Phase 1)	NCT04507971 (Phase 1), NCT03832400 (Phase 1), NCT03686202 (Phase 1)
Single microbes for gut microbiota (*n* = 10)									
Yes	Bacteria (Lactococcus lactis)	Engineered to secrete human IL‐10 and autoantigen human proinsulin	Engineered strain	Diabetes mellitus, type 1	AG019 (Precigen Actobio)	Oral	(co) Teplizumab	NCT03751007 (Phase 1b/2a)	None
Yes	Bacteria (*Escherichia coli* Nissle 1917)	Engineered to express phenylalanine‐metabolizing enzymes	Engineered strain	Phenylketonuria	SYNB1618 (Synlogic)	Oral	None	NCT04534842 (Phase 2)	None
No	Bacteria (*Enterococcus gallinarum*)	N/A	DdSS	Solid tumors	MRx0518 (4D Pharma/Merck & Co.)	Oral	(co) Pembrolizumab	NCT03637803 (Phase 1/2)	NCT03851250 (Phase 1/2), NCT04363372 (Phase 2), NCT04193904 (Phase 1), NCT03934827 (Phase 1)
No	Bacteria (Clostridium butyricum)	N/A	DdSS	Advanced kidney cancer	CBM588 (City of Hope Medical Center/National Cancer Institute)	Oral	(co) Ipilimumab and nivolumab	NCT03829111 (Phase 1)	NCT03922035 (Phase 1)
Engineered microbes for cancer (*n* = 13)									
Yes	Bacteria (*Salmonella typhimurium*)	Engineered to secrete VEGFR‐2 cDNA	Engineered strain	Recurrent glioblastoma	VXM01 (Vaximm/Merck/Pfizer)	Oral	None	NCT03750071 (Phase 1/2)	None
Yes	Bacteria (*Bifidobacteria longum*)	Engineered to secrete IL‐12	Engineered strain	Solid tumors	bacTRL‐IL‐12 (IQVIA)	IV	None	NCT04025307 (Phase 1)	None
Yes	Bacteria (*Escherichia coli*)	Engineered to express STING agonist‐producing enzymes	Engineered strain	Metastatic solid neoplasm, lymphoma	SYNB1891 (Synlogic/IQVIA)	IT	(co) Atezolizumab	NCT04167137 (Phase 1)	None
Yes	Bacteria (*Listeria monocytogenes*)	Engineered to secrete antigen‐adjuvant fusion protein for HPV	Engineered strain	Cervical cancer	ADXS11‐001 (Advaxis/Gynecologic Oncology Group)	IT	None	NCT02853604 (Phase 3)	NCT02291055 (Phase 1/2), NCT02002182 (Phase 2)
Yes	Bacteria (*Listeria monocytogenes*)	Engineered to secrete patient‐specific neoantigens	Engineered strain	Metastatic cancer	ADXS‐NEO (Advaxis)	IT	None	NCT03265080 (Phase 1)	NCT03847519 (Phase 1/2), NCT02325557 (Phase 1/2)
Yes	Bacteria (*Lactococcus lactis*)	Engineered to secrete Trefoil Factor 1	Engineered strain	Oral mucositis	AG013 (Oragenics)	Oral rinse	None	NCT03234465 (Phase 2)	None
Microbes for other applications (*n* = 6)									
No	Bacteria (Lactobacillus crispatus CTV‐05)	N/A	DdSS	Recurrent urinary tract infections	LACTIN‐V (University of Washington)	Suppository	None	NCT03151967 (Phase 2/3)	NCT03992534 (Phase 1)
No	Bacteria (*Nitrosomonas eutropha*)	N/A	DdSS	Acne vulgaris	B244 (AOBiome)	Topical spray	None	NCT02832063 (Phase 2)	NCT04490109 (Phase 2)

Abbreviations: Cell Modifications: HPV, human papillomavirus; IL, interleukin; VEGFR, vascular endothelial growth factor receptor; STING, stimulator of interferon response cGAMP interactor. Sources: DMC, defined microbe consortia; DdSS, donor‐derived single strain; UMC, undefined microbe consortia. Routes: IV, intravenous; IT, intratumoral.

Distinct from consortia‐based therapies, single strain therapies are currently in clinical trials for indications including bacterial infections, inflammatory diseases, metabolic disorders, cancer, and others. Unlike consortia‐based therapies, single strains offer more controlled and defined pathways for the discovery of specific pharmacological mechanisms of action, genetic engineering, formulation development, and consistent manufacturing, thereby reducing complexities for key regulatory considerations.^104^ Single, donor‐derived strains constitute 56% of the microbe clinical trials. Of these, 21% were rationally selected for specific therapeutic action(s) as nongenetically engineered strains (Figure 2). These therapies are delivered in a variety of formulations and via a range of administration routes and include oral capsules, topical creams and sprays, and intravaginal suppositories. To enhance the natural functions of particular microbes, genetic engineering strategies can also be used to facilitate novel functions in the gut such as the metabolism of indigestible compounds or the delivery of therapeutic proteins and peptides directly to the gastrointestinal mucosa. Genetically engineered single strains of bacteria make up 35% of the current microbe clinical trials (Figure 2) with 72% of these trials indicated for cancer treatment. The strains used in these trials are often attenuated strains of pathogenic bacteria engineered to secrete tumor‐specific antigens and are delivered via intratumoral injection.

A subset of microbe therapies in the clinic are administered in combination with immunotherapies. Based on our analysis, 57% (12/21) of all microbe‐based trials for cancer applications involve combination treatments with immunomodulatory antibodies. Aside from cancer treatment, genetically engineered single strains are also in clinical trials for other indications including diabetes (NCT03751007), oral mucositis (NCT03234465), and rare metabolic disorders (NCT04534842). While microbe‐based therapies are still in their infancy, preclinical efforts to uncover a deeper understanding of the links between the gut microbiome, cancer, and the immune system may lead to their expansion and utility in the clinic.^105^, ^106^, ^107^

## 5 MAIN CHALLENGES

The success of cell therapies will rely on the development of solutions to address critical translational challenges. Here, we focus our discussion on common translational barriers faced by the majority of cell therapies including (i) biological challenges, (ii) manufacturing challenges, and (iii) regulatory challenges. We also briefly highlight some specific, unique considerations for certain classes of cell therapy such as CAR‐T.

### 5.1 Biological challenges

There are many common challenges related to the biological activity of cell therapies. These include safety, functional heterogeneity, the maintenance of desired biological functions in vivo, and targeted delivery.

#### 5.1.1 Safety and immunogenicity

Safety limitations associated with the cell source, namely immunogenicity, are an important consideration for allogeneic therapies. This challenge is most prominent for stem cells, especially HSCs, as GvHD remains a substantial challenge in HSCT and is a major contributor to graft failure. Many HSCT post‐treatments, usually small molecules and biologics, are being investigated to prevent GvHD. Some novel agents that have progressed to Phase 3 trials include alpha‐1 antitrypsin (NCT04167514), vedolizumab (NCT03657160), and CD24Fc (NCT04095858). In addition, a combination cell therapy that uses mesenchymal stromal cells to control GvHD has also progressed to Phase 3 (NCT04629833). While immunosuppression can effectively reduce host alloreactivity, this leaves the patient susceptible to viral infections, which can induce graft‐associated morbidity and mortality. Notably, adoptive transfer of VSTs is currently being investigated to control viral infections in immunosuppressed patients.^108^ For example, transplant donor‐derived cytotoxic T lymphocytes (CTLs) are being investigated for the treatment of transplant‐related CMV in a Phase 4 trials (NCT03004261), where the cells are primed ex vivo with CMV antigen peptides before administration. The risk of GvHD is also a significant challenge for the translation of many allogeneic immunotherapies, such as T cells and DCs. On the other hand, NK cell therapy is commonly conducted using donor cells. Allogeneic NK cells do not directly induce GvHD because their cytotoxic activities are dampened by inhibitory receptors on healthy cells; therefore, they are usually associated with only minimal immunogenicity.^109^ Despite the aforementioned challenges, allogeneic therapies provide a major advantage in terms of streamlining manufacturing processes and, for some applications, producing a sufficient dose. As a result, allogeneic cells are being investigated in clinical trials covering all the cell types mentioned here. For example, in the T‐cell field, allogeneic CAR‐T therapy has the potential to decrease patient‐to‐patient heterogeneity, shorten waiting times, and reduce costs, thereby improving both efficacy and accessibility. We identified 26 trials using allogeneic anti‐CD19 CAR‐T cells, all of which are presently in Phase 1 or 1/2.

In addition to cell source‐related challenges, GM cell therapies encounter additional safety limitations. For therapies in which genetic modifications are used to induce receptor expression, risks to the patient include receptor cross‐reactivity, immunogenicity, and elaborated receptor‐mediated cytokine signaling processes.^110^ Other cells that use editing systems (e.g., CRISPR‐Cas9 and zinc finger nucleases) may encounter functional challenges associated with off‐target effects. Specifically, cells with GM receptors like CAR‐ and TCR‐T cells may cross‐react with host antigens to produce widespread off‐target, off‐tumor effects, while low‐level expression of the target on other tissues can lead to on‐target, off‐tumor effects. Cytokine release syndrome (CRS) is one major untoward effect of CAR‐T therapy. This condition is characterized by an excessive inflammatory signaling cascade, which is initiated by the adoptively transferred cells and potentiated by other immune cells like macrophages. If not rapidly treated, CRS leads to shock, neurotoxicity, and potentially organ failure and death. The incidence of CRS among patients receiving FDA‐approved CAR‐T therapy remains high, with rates greater than 50% and as high as 90% reported for Kymriah® and Yescarta®, respectively.^111^, ^112^, ^113^ CAR‐NK cells may face similar challenges, although their in vivo persistence is shorter than that of CAR‐T cells.^89^ To address CRS, additional nonreceptor genetic modifications are currently being explored to selectively eliminate adoptively transferred cells. For example, CAR‐T cells engineered with a “suicide gene” can display a molecule on their surface that specifically binds an intravenously administered cytotoxic prodrug, thereby enabling selective and tunable depletion of the CAR‐T cells in case of intolerable side effects.^110^, ^114^ Specifically, cells have been modified with truncated CD19, truncated epidermal growth factor receptor (EGFR), inducible caspase 9 (iCasp9), and herpes simplex virus thymidine kinase (HSV‐TK) suicide genes, among others. This strategy is investigated in clinical trials to prevent both CRS in the treatment of liquid and solid cancers (NCT03016377, NCT04377932) and GvHD in patients who have received HSCT (NCT00914628). For this strategy to be successful, more information is needed about the clinical parameters that warrant a prodrug infusion and the anti‐tumor efficacy postadministration.

Additional strategies for the fine‐tuning of CAR‐T functionality are currently being explored in the preclinical sphere. Many of these involve the use of inducible vectors, such as small molecules, light, and hypoxia, to control the presentation of functional CARs. For example, an inducer may switch on CAR‐T function by regulating CAR expression^115^, ^116^ or promoting receptor dimerization.^117^ Alternatively, they may disable the cells by triggering CAR degradation.^117^, ^118^ These strategies enable the dampening of overactive cells without the need to induce apoptosis, which represents a key improvement over the suicide gene systems currently used in the clinic. Logic gating is a distinct strategy that uses a combinatorial antigen‐targeting design to prevent the CAR‐T cells from becoming activated in healthy tissues, which may express low levels of the main target antigen.^119^ Instead, cytotoxic functions are only induced in cancerous tissues that overexpress a second ligand. This strategy has shown preclinical success in cases where antigen‐expressing tumor and healthy cells are spatially separated. In the future, these controllable systems may have the potential to abrogate serious toxicities while maintaining anti‐tumor efficacy in patients.

#### 5.1.2 Functional heterogeneity

Maintaining and tuning cell functionality in vivo is also a major biological challenge, especially for cells in the immunotherapy space. One general limitation is functional heterogeneity, which introduces batch‐to‐batch variation in the product and therefore patient‐to‐patient variation in the functional response. These factors add to the complexity of treating heterogeneous disease microenvironments that exist within each patient. As a result, outcomes observed in clinical trials can be highly variable. These inconsistencies make it difficult to assess translational potential and properly identify which patients might benefit the most from therapy. MSCs in particular are impacted by these discrepancies, with differences in donor sources, tissue sources, subpopulation isolation procedures, cell storage conditions, and other manufacturing processes leading to substantial variation both within and among trials.^120^, ^121^ Future approaches to address the functional heterogeneity challenge should consider both the manufacturing and clinical trial design perspectives. Better characterization and quality control of cell products during the manufacturing process will be required to maintain functional homogeneity. In addition, new manufacturing strategies, such as iPSC‐derived cell manufacturing, should be further explored.^122^ Finally, clinical trial design will need to include more comprehensive, standardized documentation of the properties of injected cells. Using these data, cross‐analyses of the relationships between cell identities/properties and safety/efficacy may be better performed to identify key attributes and inform future directions.

#### 5.1.3 Maintenance of biological activities

The efficacy of cell therapies is highly dependent on the ability of cells to retain functionality in an in vivo context. Changes in the function of living cells post‐administration, such as exhaustion and inadequate persistence, are barriers for many therapies including T cells, DCs, and NK cells. Solutions to these challenges are often complex, as simply altering the cell dose may not be feasible due to manufacturing and safety limitations. Approaches such as ex vivo cell preconditioning and combination therapy are relevant for many types of cells and are being actively explored in clinical trials. For example, MSCs can be pretreated with cytokines, small molecules, hypoxia, and/or biomaterials to tune their functionality according to the desired application.^47^, ^123^ For example, MSC preconditioning in a hypoxic environment has been shown to upregulate genes associated with survival, leading to improved persistence.^123^ Similarly, NK cell efficacy is reliant upon sufficient ex vivo expansion in the presence of cytokines such as IL‐2 and IL‐15. However, this approach is a double‐edged sword, as these cells may develop a dependency on this environment and later become exhausted or die via apoptosis in vivo.^124^ One novel expansion method is the incubation of cytotoxic NK cells with “feeder” NK cells containing membrane‐bound stimulatory cytokines. This approach enables the expansion of a sufficient dose from a single small‐volume blood draw, eliminating the need for a donor product (NCT02809092). Many NK cell clinical trials also employ combination therapies to either sustain NK cell function directly or synergize with the therapy. For example, anti‐tumor antibodies may improve NK cell retention in solid tumors, while co‐administered cytokines help maintain cytotoxic activities. Synergistic effects have been observed with the co‐administration of NK cells and checkpoint blockade antibodies (NCT03958097). Likewise, the biological activity of DC therapies is dependent on multiple factors including the maturation process, source, loading method, and antigen target(s). DC therapies may also benefit from combination approaches to overcome immunosuppressive microenvironments and enhance in vivo activity. For instance, the administration of proinflammatory cytokines and Toll‐like receptor (TLR) agonists at the injection site has proven effective for the enhancement of DC migration into the lymph nodes.^125^

Similar to NK cells and DCs, T cells have also benefited from combination strategies, especially checkpoint blockade and low‐dose IL‐2. CAR‐T cells in particular have demonstrated improved activation and persistence with the incorporation of immunomodulatory features via genetic modification. The addition of costimulatory domains to the intracellular portion of the CAR was one of the earliest approaches used to prevent cell exhaustion induced by chronic stimulation with the target antigen. More recently, the secretion or display of therapeutic payloads directly from the delivered cells has emerged as a promising approach in the clinical landscape.^126^ There are many current trials employing CAR‐T cells that have been modified to display or secrete (i) checkpoint inhibitors to prevent T‐cell exhaustion or (ii) cytokines (e.g., IL‐15, IL‐12, IL‐7) to promote T cell persistence and proliferation in an autoregulatory manner. In the treatment of solid tumors, these cytokines can also promote a Th1 environment to overcome immunosuppression.^110^, ^127^ Two of these trials have progressed to Phase 2, one with cells that secrete a mutant PD‐1 fusion protein (NCT04162119), and another with cells that secrete IL‐7 and CCL19 (NCT03929107). Comparable genetic modifications have also been used in the stem cell field, albeit less frequently. For example, MSCs have been engineered to express therapeutic payloads like the cytokine TRAIL (NCT03298763). While GM cells are modified to secrete or express a payload, non‐GM cells have also been used as drug delivery vehicles and seen some successes in preclinical settings. In such cases, therapeutic cells can carry nano‐ or micro‐scale biomaterials that contain stimulatory molecules such as cytokines. This particular strategy, also called cytokine backpacking, has potential to offer a versatile alternative to genetic modification in the clinical setting.^128^, ^129^, ^130^ Currently, there is a Phase 1 trial underway for the use of IL‐15 superagonist‐loaded T cells in the treatment of solid and liquid cancers (NCT03815682).

#### 5.1.4 Delivery challenges

Targeted delivery is another biological challenge for many cell therapies, particularly those designed to treat diseases in solid tissues. Both cell migration to and localization at the target site are important considerations, and these factors may depend heavily on the initial delivery route. For example, intravenously administered T cells must sense biological cues, such as chemokine gradients and endothelial markers, to extravasate into a solid tumor. One major approach that is being explored to improve delivery is altering the administration route. CAR‐T cells indicated for solid cancers are currently administered via a variety of routes (see Section 4) to accomplish direct delivery into target tissue, a nearby artery, or cerebrospinal fluid (for neurologic tumors). New NK cell administration routes, including intracranial and intratumoral, are also being explored for the treatment of glioblastoma (NCT03383978, NCT04489420). While T and NK cells must reach the site of a tumor or infection, DCs must reach the lymph nodes to execute their antigen‐presenting functions. Injection routes including intradermal, intranodal, and subcutaneous are currently being explored to improve lymph node trafficking.^131^ For HSCs, intra‐bone infusions are currently being explored as an alternative to IV administration to speed HSC delivery and graft reconstitution in the bone marrow.^132^ While the outcomes of this approach are not yet conclusive, this administration route may reduce acute GvHD, providing a solution to this longstanding biological challenge.

### 5.2 Manufacturing challenges

The development of closed, automated manufacturing processes is a necessary yet challenging endeavor for the production of safe, high‐quality cell therapies. Current barriers and advancements related to the manufacturing of cell therapies have been reviewed in detail elsewhere.^14^, ^133^, ^134^ Briefly, the major steps in the manufacture of a relatively complex cell therapy (e.g., an autologous GM cell therapy) are as follows. First, the cells are extracted from the patient and shipped to the site of manufacture, where relevant populations are isolated. Cells are activated, genetically modified, and expanded before undergoing post‐processing, such as washing and purification. As more patient data becomes available, heightened attention should be paid to the consequences of impurities in cell therapy products. Although adverse events are rare, in one documented circumstance, the errant transduction of a single B cell with a CAR rendered CAR‐T therapy (Kymriah) ineffective.^135^ After samples are taken for quality assurance (QA) measures, the cells are concentrated and packaged by dose before being shipped to the site where the therapy will be administered.^133^ The process for allogeneic manufacturing is streamlined by the exclusion of the first extraction and shipping steps. Multiple challenges exist at each step in this workflow, though future technologies have shown promise for potential integration into a closed, automated process. Current areas of focus include cryopreservation, cell selection and activation workflows, automated batch monitoring for QA purposes, and the adoption of electronic records systems.^126^, ^134^, ^136^ These interventions have the potential to decrease the heterogeneity of cell therapies and reduce production time. In the future, decentralization of the manufacturing process will expand the global scale of cell therapies to reach patients who are currently inaccessible.

Another closely related factor is the cost associated with developing, manufacturing, and distributing cell therapies. The steep price tags of some therapies, such as CAR‐T therapy Kymriah's list price of $475,000,^137^ have drawn criticism due to concerns about patient accessibility. Advances in manufacturing procedures, particularly automation, along with widespread adoption of allogeneic therapies may help drive down direct costs in the future.

### 5.3 Regulatory challenges

In addition to the aforementioned considerations, future advancements in the clinical translation of cell therapies will rely on the development of industry‐wide and regulatory approval standards. As of 2020, there is no universal system for the classification of cell therapies. Not only does this insufficiency impede the regulatory approval process, but it also allows for a substantial degree of ambiguity in the reporting of quality attributes and clinical outcomes.^138^ In light of these concerns, the FDA released a warning regarding the dangers of unapproved stem‐cell therapies in 2020.^139^ Notably, regulatory standards related to product purity are rapidly changing in the more recently developed field of microbe‐based therapy. In early 2020, alerts were issued regarding the risks of transmission of pathogenic microorganisms and SARS‐CoV‐2 via FMT.^140^ Given the rapid emergence of many new cell therapies with novel features, it is imperative that products be categorized in a central location for the benefit of the research, medical, and patient communities. In addition, this endeavor will enable more comprehensive future studies to identify associations between cell properties and clinical efficacy. These studies may then inform decision‐making at the regulatory, clinical, and preclinical levels to accelerate the development of novel therapies. Eventually, these efforts may enable the standardization of critical quality attributes, which has the potential to streamline the development and approval processes.

## 6 CONCLUSION

Cells as living entities have unique properties and are being leveraged as potent therapies to treat diseases in a way that conventional therapies cannot. Many cell therapies have been approved by the FDA, EMA, and other regulatory agencies around the world for the treatment of many diseases. Particularly, many of these approved products have demonstrated promising efficacy in treating diseases that were thought to be incurable by conventional therapies, such as CAR‐T therapy for B‐cell malignancies. Great efforts are also being devoted to further expanding the clinical application of these approved products in other indications. In addition, with improved understanding of the biology of different living cells, the clinical landscape is seeing a number of clinical trials investigating newer types of cell therapies like microbes. These efforts are not only focusing on exploring more types of cells that have unique biological functions, but further expanding the spectrum of indications cell therapies can treat. Still, cell therapies are in their early stages of development and face biological, manufacturing, and regulatory challenges that affect their clinical translation. Solving these challenges will need to involve united efforts including further fundamental biological studies, standardized clinical listing and data sharing protocols, and automated, decentralized manufacturing capabilities. However, with its proven clinical success and ongoing clinical advances, cell therapy remains a highly active area of research and more disruptive cell therapy products are expected to emerge in the near future.

## AUTHOR CONTRIBUTIONS

**Li Wen Wang:** Data curation; formal analysis; investigation; visualization; writing‐original draft; writing‐review & editing. **Morgan Janes:** Conceptualization; formal analysis; methodology; visualization; writing‐original draft; writing‐review & editing. **Ninad Kumbhojkar:** Conceptualization; formal analysis; writing‐original draft; writing‐review & editing. **Neha Kapate:** Data curation; formal analysis; writing‐original draft; writing‐review & editing. **John Clegg:** Data curation; formal analysis; writing‐original draft; writing‐review & editing. **Supriya Prakash:** Formal analysis; methodology; visualization; writing‐original draft. **Mairead Heavey:** Formal analysis; writing‐original draft. **zongmin Zhao:** Conceptualization; formal analysis; methodology; writing‐original draft; writing‐review & editing. **Aaron Anselmo:** Conceptualization; data curation; formal analysis; funding acquisition; visualization; writing‐original draft; writing‐review & editing. **Samir Mitragotri:** Conceptualization; funding acquisition; writing‐review & editing.

## Supporting information


**Supplementary Table 1** Abbreviation list.
**Supplementary Table 2.** Clinically approved cell therapies being investigated for additional indications in current clinical trials.
**Supplementary Table 3.** Antigen targets in CAR‐T cell trials. Antigen names are listed as outlined in clinical trial data. Alternative identifiers are shown in parentheses. Viral targets are grouped and listed by virus name (ex. HIV) as opposed to specific antigen.
**Supplementary Table 4.** Antigen targets in TCR‐T cell trials. Antigen names are listed as outlined in clinical trial data. Viral targets are grouped and listed by virus name (ex. HPV) as opposed to specific antigen.
**Supplementary Table 5.** Viral targets in virus‐specific T cell trials.
