# Supplementary material for: Cell therapies in the clinic
Source: Bioeng Transl Med. 2021 Feb 26;6(2):e10214. doi: 10.1002/btm2.10214 (PMC8126820; doi:10.1002/btm2.10214)
Supplement: Supplementary file 1 — Supplementary Table 1 Abbreviation list. Supplementary Table 2. Clinically approved cell therapies being investigated for additional indications in current clinical trials. Supplementary Table 3. Antigen targets in CAR‐T cell trials. Antigen names are listed as outlined in clinical trial data. Alternative identifiers are shown in parentheses. Viral targets are grouped and listed by virus name (ex. HIV) as opposed to specific antigen. Supplementary Table 4. Antigen targets in TCR‐T cell trials. Antigen names are listed as outlined in clinical trial data. Viral targets are grouped and listed by virus name (ex. HPV) as opposed to specific antigen. Supplementary Table 5. Viral targets in virus‐specific T cell trials. [file BTM2-6-e10214-s001.docx]

**Supporting Information**

**Cell Therapies in the Clinic**

Lily Li-Wen Wang,^1,2,3^ Morgan E. Janes,^1,2,3^ Ninad Kumbhojkar,^1,2,*^ Neha Kapate,^1,2,3,*^ John R. Clegg,^1,2^  Supriya Prakash,^1,2^ Mairead K. Heavey,^4^ Zongmin Zhao,^1,2^ Aaron C. Anselmo,^4,†^ and Samir Mitragotri^1,2,†^

^1^ John A. Paulson School of Engineering & Applied Sciences, Harvard University, Cambridge, MA 02138, USA

^2^ Wyss Institute for Biologically Inspired Engineering, Boston, MA 02115, USA

^3^ Harvard-MIT Division of Health Sciences and Technology, Massachusetts Institute of Technology, Cambridge, MA 02139, USA

^4^ Division of Pharmacoengineering and Molecular Pharmaceutics, Eshelman School of Pharmacy, University of North Carolina at Chapel Hill, Chapel Hill, NC 27599, USA

^†^Corresponding author

Email: [aanselmo@email.unc.edu](mailto:aanselmo@email.unc.edu)

Email: [mitragotri@seas.harvard.edu](mailto:mitragotri@seas.harvard.edu)

*These authors contributed equally

**Supplementary Table 1.** Abbreviation list.

| Abbreviation | Full Name |
| --- | --- |
| AD | autoimmune diseases |
| ADA | adenosine deaminase |
| ADA-SCID | adenosine deaminase deficiency |
| ADV | adenovirus |
| aGvHD | acute GvHD |
| AIDS | acquired immunodeficiency syndrome |
| ALL | acute lymphocytic leukemia |
| ALS | amyotrophic lateral sclerosis |
| AML | acute myeloid leukemia |
| APCs | antigen-presenting cells |
| ARDS | acute respiratory distress syndrome |
| Bcl11a | B-cell leukemia/lymphoma 11a |
| BCMA | B cell maturation antigen |
| BD | blood disorders |
| BD w/ RBCs | non-malignant blood disorders associated with red blood cells |
| BD w/ WBCs | non-malignant blood disorders associated with white blood cells |
| BMDSC | bone-marrow-derived stem cell |
| BMMC | bone marrow-derived mononuclear cell |
| CAR | chimeric antigen receptor |
| CBMC | cord blood mononuclear cell |
| CCR5 | C-C chemokine receptor type 5 |
| CD | cardiovascular diseases |
| CD | cluster of differentiation |
| CD19t | truncated CD19 |
| CDSCO | Central Drugs Standard Control Organization, aka Indian FDA |
| CEA | carcinoembryonic antigen |
| CFIA | Canadian Food Inspection Agency |
| CIK | cytokine-induced killer cell |
| CML | chronic myeloid leukemia |
| CMV | cytomegalovirus |
| CNS | central nervous system |
| COPD | chronic obstructive pulmonary disease |
| COVID-19 | coronavirus disease 2019 |
| CRS | cytokine release syndrome |
| CTC | circulating tumor cell |
| CTL | cytotoxic T lymphocyte |
| DCGI | Drug Controller General of India |
| DC | dendritic cell |
| DD | degenerative disease |
| DdSS | donor-derived single strain |
| DMC | defined microbe consortia |
| DOCK8 deficiency | dedicator of cytokinesis 8 deficiency |
| EBV | Epstein-Barr virus |
| EGFR | epidermal growth factor receptor |
| EMA | European Medicines Agency |
| EPC | endothelial progenitor cell |
| FMT | fecal microbiota transplant |
| FOB | fiberoptic bronchoscopy |
| GM | genetically modified |
| GM-CSF | granulocyte macrophage colony-stimulating factor |
| GPC3 | glypican 3 |
| GvHD | graft versus host diseases |
| HA-1 | minor histocompatibility antigen |
| hALDP | human adrenoleukodystrophy protein |
| HER2 | human epidermal growth factor receptor 2 |
| HIV | human immunodeficiency virus |
| HLA | human leukocyte antigen |
| HPV | human papillomavirus |
| HSC | hematopoietic stem cell |
| HSCT | hematopoietic stem cell transplant |
| HSV-TK | herpes simplex virus thymidine kinase |
| IA | intra-articular |
| IAe | intra-arterial |
| IC | intracavitary |
| iCasp9 | inducible caspase 9 |
| ICe | intracerebral |
| ICI | intracoronary infusion |
| ICr | intracranial |
| ID | infectious diseases |
| ID | intradermal |
| IDD | immune deficiency disorders |
| IFN | interferon |
| IL-15 | interleukin 15 |
| IL-2 | interleukin-2 |
| IL13Ra2 | interleukin-13 receptor alpha 2 |
| IN | intranodal |
| IP | intraperitoneal |
| iPSC | induced pluripotent stem cell |
| IT | intratumoral |
| ITh | intrathecal |
| IV | intravenous |
| IVN | intraventricular |
| JMHW | Japanese Ministry of Health and Welfare |
| KFDA | Korea Food & Drug Administration |
| KIR | killer-immunoglobulin receptor |
| LAD | leukocyte adhesion disorder |
| LSC | limbal stem cell |
| MAGE-A10 | melanoma-associated antigen 10 |
| MAGEA4 | melanoma-associated antigen 4 |
| MAPC | multipotent adult progenitor cell |
| MCL | mantle cell lymphoma |
| MD | metabolic disorders |
| MDS | myelodysplastic syndrome |
| MHC | major histocompatibility complex |
| MS | multiple sclerosis |
| MSC | mesenchymal stem cell |
| N/A | non applicable |
| NaID | non-autoimmune inflammatory diseases |
| NGM | non-genetically modified |
| NK | natural killer |
| NK-92 | immortal NK cell line |
| NKG2DL | natural killer group 2D ligand |
| NKT | natural killer T cell |
| NSC | neural stem cell |
| NY-ESO-1 | New York esophageal squamous cell carcinoma-1 |
| P. vivax | *Plasmodium vivax* |
| PAP | prostatic acid phosphatase |
| PBMC | peripheral blood mononuclear cell |
| PCD | percutaneous catheter |
| PD-1 | programmed cell death protein 1 |
| PD-L1 | programmed death-ligand 1 |
| pp65-LAMP mRNA | phosphoprotein 65 lysosomal associated membrane protein messenger RNA |
| PRAME | preferentially expressed antigen in melanoma |
| QA | quality assurance |
| r/r | relapsed or refractory |
| r/r B-cell ALL | r/r B-cell precursor acute lymphoblastic leukemia |
| RBC | red blood cell |
| Rev | regulator of expression of virion proteins |
| RhD | Rhesus D |
| RIC | reduced intensity conditioning |
| SARS-CoV-2 | severe acute respiratory syndrome coronavirus 2 |
| SC | subcutaneous |
| scFv | single-chain variable fragment |
| SCID | severe combined immunodeficiency |
| siRNA | small interfering RNA |
| SR-aGvHD | steroid-refractory acute GvHD |
| SSX | synovial sarcoma X chromosome breakpoint |
| STING | stimulator of interferon response cGAMP interactor |
| TAA | tumor-associated antigen |
| TAA-T | tumor-associated antigens T cells |
| Tat | transactivator of expression |
| TCR | T cell receptor |
| Th1 | T helper cell type 1 |
| Th2 | T helper cell type 2 |
| TIL | tumor-infiltrating lymphocyte |
| TLR | Toll-like receptor |
| TRAIL | TNF-related apoptosis-inducing ligand |
| TrD | transplant-related diseases |
| TrDG | transplant-related disorders - graft versus host diseases |
| TrDI | transplant-related disorders - infection |
| Treg | regulatory T cell |
| TT-RNA | total tumor RNA |
| UMC | undefined microbe consortia |
| Unk | unknown |
| USFDA | the United States Food and Drug Administration |
| VEGFR | vascular endothelial growth factor receptor |
| VST | virus-specific T cell |
| WAS | Wiskott-Aldrich syndrome |
| WT1 | Wilms Tumor antigen 1 |

**Supplementary Table 2.** Clinically approved cell therapies being investigated for additional indications in current clinical trials.

| *Name / Trade Name (Manufacturer)* | | *Investigated Indications* | [*ClinicalTrials.gov*](http://clinicaltrials.gov/) *Identifier Trial Status* |
| --- | --- | --- | --- |
| *T Cell* | | | |
| Tisagenlecleucel / Kymriah^®^ (Novartis) | | Various liquid cancers: primary CNS lymphoma, r/r primary CNS lymphoma, follicular lymphoma, recurrent mantle cell lymphoma, acute biphenotypic leukemia, minimal residual disease, small lymphocytic lymphoma, stage III & IV chronic lymphocytic leukemia | 20 studies:  4 Not yet recruiting; 11 Recruiting; 5 Active |
| Axicabtagene ciloleucel / Yescarta^®^ (Kite) | | Various liquid cancers: follicular lymphoma, marginal zone lymphoma, indolent non-Hodgkin lymphoma, primary mediastinal (thymic) large B-Cell lymphoma, transformed follicular lymphoma to Diffuse large B-cell lymphoma, mantle cell lymphoma | 12 studies:  2 Not yet recruiting; 4 Recruiting; 6 Active |
| Brexucabtagene autoleucel / Tecartus™ (Kite) | Various liquid cancers: r/r chronic lymphocytic leukemia, r/r small lymphocytic lymphoma, r/r mantle cell lymphoma, r/r non-Hodgkin lymphoma | 4 studies:  2 Recruiting; 2 Active | |
| N/A / ImmunCell-LC^®^ (Green Cross Cell) | Hepatocellular carcinoma | 2 Recruiting | |
| *Stem Cell - HSC* | | | |
| HPC, Cord Blood / Hemacord™ (New York Blood Center) | Acute ischemic stroke | 1 not yet recruiting | |
| Betibeglogene autotemcel / Zynteglo™ (bluebird bio) | Beta thalassemia major, sickle cell disease | 4 studies  2 Recruiting; 2 Active | |
| *Stem Cell - MSC* | | | |
| N/A / Cellgram™ (Pharmicell) | Acute myocardial infarction, alcoholic liver cirrhosis, spinal cord Injury, erectile dysfunction | 4 Recruiting | |
| Darvadstrocel / Alofisel^®^ (TiGenix NV/ Takeda) | Complex perianal fistulas in Crohn’s disease | 3 studies:  2 Recruiting; 1 Active | |
| Remestemcel-L / Prochymal^®^ (Osiris Therapeutics/ Mesoblast Limited) | ARDS, COVID-19, ulcerative colitis | 3 Recruiting | |
| N/A / Cartistem^®^ (Medipost) | Chondral or osteochondral lesion of talus | 1 Enrolling by invitation | |
| *Stem cell - Other* | | | |
| N/A / Holoclar^®^ (Chiesi) | Limbal stem cell deficiency | 1 Active | |
| *Dendritic Cell* | | | |
| Sipuleucel-T / Provenge^®^ (Dendreon) | Prostate cancer | 5 Active | |

Abbreviations:

***Indications****:* Acute respiratory distress syndrome (ARDS), coronavirus disease 2019 (COVID-19)

**Supplementary Table 3.** Antigen targets in CAR-T cell trials. Antigen names are listed as outlined in clinical trial data. Alternative identifiers are shown in parentheses. Viral targets are grouped and listed by virus name (ex. HIV) as opposed to specific antigen.

| Target | # of Trials | % |
| --- | --- | --- |
| CD19 | 226 | 36.9 |
| BCMA (CD269) | 65 | 10.6 |
| CD22 | 38 | 6.2 |
| CD20 | 27 | 4.4 |
| CD123 | 19 | 3.1 |
| GD2 | 18 | 2.9 |
| Mesothelin | 18 | 2.9 |
| CD30 | 14 | 2.3 |
| CD38 | 10 | 1.6 |
| HER2 | 10 | 1.6 |
| CD7 | 9 | 1.5 |
| GPC3 | 9 | 1.5 |
| CD33 | 8 | 1.3 |
| EGFR | 8 | 1.3 |
| MUC1 | 7 | 1.1 |
| PSMA | 7 | 1.1 |
| CEA | 6 | 1 |
| NKG2DL | 6 | 1 |
| CLL1 | 5 | 0.8 |
| CS1 (SLAMF7) | 5 | 0.8 |
| B7H3 (CD276) | 5 | 0.8 |
| CD138 | 4 | 0.7 |
| CD70 | 4 | 0.7 |
| IL-13Rα2 | 4 | 0.7 |
| NY-ESO-1 | 3 | 0.5 |
| CD4 | 3 | 0.5 |
| CD44v6 | 3 | 0.5 |
| CD5 | 3 | 0.5 |
| Claudin 18.2 | 3 | 0.5 |
| EpCAM | 3 | 0.5 |
| PSCA | 3 | 0.5 |
| LeY | 3 | 0.5 |
| AXL | 2 | 0.3 |
| c-Met | 2 | 0.3 |
| CD10 (gp100) | 2 | 0.3 |
| CD133 | 2 | 0.3 |
| CD56 | 2 | 0.3 |
| CD147 | 2 | 0.3 |
| DR5 | 2 | 0.3 |
| FRα | 2 | 0.3 |
| ROR2 | 2 | 0.3 |
| HIV | 2 | 0.3 |
| Kappa immunoglobulin | 2 | 0.3 |
| MUC16 | 2 | 0.3 |
| CD117 | 1 | 0.2 |
| CD135 | 1 | 0.2 |
| Integrin β7 | 1 | 0.2 |
| CD171 | 1 | 0.2 |
| CCR4 | 1 | 0.2 |
| CD37 | 1 | 0.2 |
| EphA2 | 1 | 0.2 |
| ErbB | 1 | 0.2 |
| FAP | 1 | 0.2 |
| ICAM-1 | 1 | 0.2 |
| MAGE-A1 | 1 | 0.2 |
| MAGE-A4 | 1 | 0.2 |
| MPP2 | 1 | 0.2 |
| Nectin-4 | 1 | 0.2 |
| ROBO1 | 1 | 0.2 |
| ROR1 | 1 | 0.2 |
| T2C | 1 | 0.2 |
| TGF-β | 1 | 0.2 |
| TnMUC1 | 1 | 0.2 |
| TRBC1 | 1 | 0.2 |
| Unknown/other | 12 | 2 |

**Supplementary Table 4.** Antigen targets in TCR-T cell trials. Antigen names are listed as outlined in clinical trial data. Viral targets are grouped and listed by virus name (ex. HPV) as opposed to specific antigen.

| Target | # of Trials | % |
| --- | --- | --- |
| NY-ESO-1 | 13 | 17.1 |
| EBV | 11 | 14.5 |
| MAGE-A4 | 5 | 6.6 |
| AFP | 5 | 6.6 |
| HPV | 5 | 6.6 |
| CD19 | 5 | 6.6 |
| HBV | 3 | 3.9 |
| WT1 | 3 | 3.9 |
| MAGE-A3 | 2 | 2.6 |
| Tyrosinase | 2 | 2.6 |
| KRAS G12D | 2 | 2.6 |
| HA-1 | 2 | 2.6 |
| Mesothelin | 2 | 2.6 |
| MAGE-A10 | 2 | 2.6 |
| PRAME | 2 | 2.6 |
| KRAS G12V | 1 | 1.3 |
| MCPyV | 1 | 1.3 |
| MART-1 | 1 | 1.3 |
| HERV-E antigen | 1 | 1.3 |
| LAGE-1a | 1 | 1.3 |
| MAGE-A1 | 1 | 1.3 |
| MAGE-A8 | 1 | 1.3 |
| MAGE-A6 | 1 | 1.3 |
| HIV | 1 | 1.3 |
| Unknown/ other | 3 | 3.9 |

**Supplementary Table 5.** Viral targets in virus-specific T cell trials.

| Target | # of Trials | % |
| --- | --- | --- |
| CMV | 23 | 29.1 |
| EBV | 21 | 26.6 |
| ADV | 11 | 13.9 |
| BKV | 7 | 8.9 |
| SARS-CoV-2 | 3 | 3.8 |
| HIV | 3 | 3.8 |
| HBV | 1 | 1.3 |
| HPV | 1 | 1.3 |
| HHV6 | 1 | 1.3 |
| JV | 1 | 1.3 |
| HPIV3 | 1 | 1.3 |
| Unknown | 6 | 7.6 |
